# Prior context influences motor brain areas in an auditory oddball task and prefrontal cortex multitasking modelling

**DOI:** 10.1186/s40708-021-00124-6

**Published:** 2021-03-21

**Authors:** Carlos A. Mugruza-Vassallo, Douglas D. Potter, Stamatina Tsiora, Jennifer A. Macfarlane, Adele Maxwell

**Affiliations:** 1 Grupo de Investigación de Computación Y Neurociencia Cognitiva, Facultad de Ingeniería Y Gestión, Universidad Nacional Tecnológica de Lima Sur – UNTELS, Lima, Perú; 2 Neuroscience and Development Group, Arts and Science, University of Dundee, Dundee, UK; 3 School of Psychology, University of Lincoln, Lincoln, United Kingdom; 4 NHS Tayside, Ninewells Hospital, Dundee, UK

**Keywords:** Attention, Cognitive modelling, Electroencephalography (EEG), Event-related potential (ERP), Executive function, Cue–target onset asynchrony (CTOA), Functional magnetic resonance imaging (fMRI), Motor networks, Multitask applications, Prefrontal cortex (PFC), Orienting of attention, Running average reaction times

## Abstract

In this study, the relationship of orienting of attention, motor control and the Stimulus- (SDN) and Goal-Driven Networks (GDN) was explored through an innovative method for fMRI analysis considering all voxels in four experimental conditions: standard target (Goal; G), novel (N), neutral (Z) and noisy target (NG). First, average reaction times (RTs) for each condition were calculated. In the second-level analysis, ‘distracted’ participants, as indicated by slower RTs, evoked brain activations and differences in both hemispheres’ neural networks for selective attention, while the participants, as a whole, demonstrated mainly left cortical and subcortical activations. A context analysis was run in the behaviourally distracted participant group contrasting the trials immediately prior to the G trials, namely one of the Z, N or NG conditions, i.e. Z.G, N.G, NG.G. Results showed different prefrontal activations dependent on prior context in the auditory modality, recruiting between 1 to 10 prefrontal areas. The higher the motor response and influence of the previous novel stimulus, the more prefrontal areas were engaged, which extends the findings of hierarchical studies of prefrontal control of attention and better explains how auditory processing interferes with movement. Also, the current study addressed how subcortical loops and models of previous motor response affected the signal processing of the novel stimulus, when this was presented laterally or simultaneously with the target. This multitasking model could enhance our understanding on how an auditory stimulus is affecting motor responses in a way that is self-induced, by taking into account prior context, as demonstrated in the standard condition and as supported by Pulvinar activations complementing visual findings. Moreover, current BCI works address some multimodal stimulus-driven systems.

**Supplementary Information:**

The online version contains supplementary material available at 10.1186/s40708-021-00124-6.

## Introduction

Recent works in stimulus-driven neural networks and learning systems are awakening the interest in multimodal attention systems, such as works in Brain Computer Interface (BCI) systems in both visual and auditory modalities [1, 2], also considering tasks with multiple conditions [3]. In the present work, the interaction of the auditory and motor systems is studied and modelled using an odd/even auditory number decision task, whilst performing simultaneous scalp electroencephalography (EEG) and functional magnetic resonance imaging (fMRI) recordings. The effects of prior context on attention have traditionally been studied with fMRI in visual tasks. Koechlin and colleagues [4, 5] used an experimental task in which participants were asked to discriminate coloured shapes or letters, or ignore a non-goal stimulus, on the basis of an instruction cue that initiated each block. Based on their findings, they suggested that the lateral frontal lobes contribute to a cascade of control processes mediating sensory, contextual, and episodic control, implemented in premotor, caudal and rostral lateral prefrontal cortical regions, respectively [4]. Therefore, pending behavioural responses are maintained and managed by prefrontal areas, and the activation of frontal areas can be affected by multitasking. On the other hand, it has been previously shown [3] that in an auditory oddball task with 4 conditions where participants had to maintain a number parity decision goal while ignoring novel distractors, stimulus properties (here features) and previous context were covariates that helped to understand and explain attention switching. Furthermore, it was shown that these findings were not related to the time intervals between stimuli, as measured by the P300. Their study pointed to single-trial event-related potential (ERP) dependence on prior signals; the longer the signals in time, the fewer effects mismatch negativity (MMN) and the stimulus features explained about the variance of the P300 amplitude. Moreover, a similar study that used linear filtering suggested a use for a schizophrenia therapy study of attention and executive functions [6]. The addition of fMRI measures in this type of research would be beneficial in better understanding how prior context influences behavioural response in the auditory modality.

Current theories of attention assume the involvement of a distributed control network of areas in stimulus-driven selection of the behaviourally relevant information [7]. Furthermore, these control networks share common areas and interact with the goal-driven network (GDN) (see review of the fronto-parietal visual attention network using single-cell recordings in monkeys and fMRI in humans by Kastner and Ungerleider [8]). Moreover, the actor critic architecture for learning and execution proposed by Savalia and colleagues [9] where time and hierarchical management of sequence induce different work at loop on basal ganglia–frontal cortex and hippocampus–frontal cortex. While other studies were seeking how motor responses were done differently for every participant in a decision-making task [10] and how the reference frame is important for decisions in hand choice [11]. Even more, on goal-driven tasks behavioural motor answers that used greater auditory processing suppress responses in the auditory cortex [12] and recent report has shown motor-dependent changes in auditory cortical dynamics were driven by a subset of neurons in the secondary motor cortex that innervate the auditory cortex and are active during movement [13]. These studies have led to the uncertainty of how the auditory stimulus is affecting motor responses in a kind of self-induced motor signal [14]. The present work has looked at motor responses and their relation with prefrontal areas.

Wagner and colleagues used a word goal decision task to find how some tasks are recognized or not in the human brain. The goal was a semantic signal (abstract or concrete) and a non-semantic signal (upper or lower-case letter). Results pointed to the lateralization response for the left prefrontal cortex, left fusiform gyrus and temporal cortices [9]. However, this study focused on explaining how lateralization activations may be changing in time for each stimulus type.

Few studies have explored the generators of auditory novelty using EEG and fMRI measures. Opitz and colleagues [15] used a block design in an auditory oddball task, where the goal standard stimulus was a tone of 600 Hz (83.4%), the non-goal deviant stimulus was a tone of 1000 Hz (8.3%) and the non-goal novel stimulus was an environmental sound. They found that novel sounds activated the superior parietal cortex and those subjects showing strong N4 deflections showed an additional right prefrontal cortex (rPFC) activation [15]. Bearing in mind the distributed areas for attention [7], Strobel and colleagues aimed to improve Opitz and colleagues [15] study using simultaneous EEG/fMRI recordings with an event-related design in an auditory oddball task. They used tones of 350 and 650 Hz and environmental sounds where participants were required to silently count standard tones as targets in 50% of the cases and novel sounds as targets in the other 50%. They found that the bilateral superior temporal and right inferior frontal areas showed strongest activation with novel sounds [16].

Kiehl and colleagues used fMRI to study the brain areas activated in an auditory oddball task seeking to answer whether gender influences the magnitude or distribution of brain activity associated with the P3a and P3b responses. They implemented a task in which the standard tone stimulus had a probability of 0.8, the target tone stimulus had a probability of 0.1 and the novel stimuli had a probability of 0.1 with an Inter-Trial Interval (ITI) of 2000 ms. They examined hemodynamic fMRI responses of target detection and novel stimulus processing in five groups of 20 subjects. They did not find evidence of a gender effect, but this study is relevant to the present research because it was an oddball task, and the ITI was similar. We used a single sound per trial and gender was imbalanced. They found around 28 brain areas for the target over the standard stimulus (the superior parts of the left PreCentral Gyrus, left middle and Inferior Frontal Gyrus, and brainstem), 20 brain areas for the novel over the non-goal standard tone stimulus (bilateral Amygdala, Anterior and Posterior Cingulate, bilateral inferior parietal lobe, and brainstem), 29 brain areas for the target over the novel stimulus (bilateral middle Frontal Gyrus, right Inferior Frontal Gyrus, left PreCentral and postcentral Gyrus, and right Cerebellum) and 29 brain areas for the target over the novel stimulus (bilateral middle Frontal Gyrus, bilateral middle Temporal Gyrus, and right precentral Gyrus and additional regions in left middle frontal Gyrus, right middle temporal Gyrus, and left angular Gyrus and Precuneus) [17]. Therefore, in terms of comparison we should expect to find several areas activated for the four switching conditions.

Nowadays, mixed modalities are tough to cognitive robotics, many works employ image recognition in combination to motor answers due to its many potential applications. For example Zeng and colleagues, employ several paths (Somatosensory Input → Thalamus → Primary somatosensory cortex → SMG), the visual ventral stream (Visual Input → Thalamus → Primary visual cortex → EBA/OFA → FBA/FFA → ITG → SMG), and the visual dorsal stream [Visual Input → Thalamus → Primary visual cortex → (MT/V5, EBA/OFA, FBA/FFA) → STS → SMG [18] without employing auditory components for cognitive tasks.

On the one hand, some multisensorial modalities, such as visuo-haptic object recognition are now as multimodal interactions take place between the two sensory modalities [12]. Evolutionary multitasking was recently developed through algorithms seeking brain function. This multi-X evolutionary computation is based on multi-objective optimization problems (MOPs) employing frequency or objective functions (f) for vectors of decision variables (y) in the search space (Y) [19] following (1):

1 $${\text{maximize}}(y \in Y) \, f\left( y \right) = \left| {f_{1} \left( y \right) \, ; \, f_{2} \left( y \right) \, ; \ldots ; \, f_{K} \left( y \right)} \right|.$$

Then, for *K* different tasks (T1, T2,… TK) the MOP in terms of the populations, multitask would follow (2), having *Σwjk* = *1,∀k; and wjk* ≥ *0; ∀j; k*

2 $$\begin{array}{*{20}c} {} \\ {{\text{maximize}}(y \in Y) } & { \sum_{k = 1}^K \int z f_{k} \left( z \right) \cdot } & {\left[ {\sum_{k = 1}^K w_{jk} \cdot p_{j} \left( z \right)} \right] \cdot {\text{ d}}z} \\ {\left\{ {wjk.pj\left( z \right)} \right\} } \\ \end{array}$$

The aims of the present analysis and modelling on the present work are to determine if the simultaneous EEG and fMRI recordings can provide insights into (a) the effect of prior stimulus contexts across participants; (b) the sources of the generators of the positive deflections in the ERP waveforms, including the smaller right lateralized positive deflection observed to novel sounds; (c) the modelling of multimodal stimulus-driven network for practical use.

Based on the findings of the literature summarized above and the results of four task switching [3], the following hypotheses were drawn for an experiment to develop a better approach for modelling:

H1: The participants must orient their attention in response to novel distractors and this should be associated with bilateral activations of the goal-driven system. This would confirm the sensitivity of the task in the framework of the distributed control of attention proposed by Corbetta and colleagues [7, 20].

H2: Bearing in mind the contextual effect of the immediately previous trial, in a task with several conditions [4, 5] several significant different brain areas should appear in different fMRI contrasts. Therefore, based on Koechlin’s findings and results in the experiment with 4 conditions [3], the Goal-driven experiment should produce significant modulations of activations in memory areas as a result of modulation by different areas of the prefrontal cortex, dependent of the level of contextually based executive controls outlined by Koechlin et al. [4, 5]. The differing contextual conditions associated with the different experimental conditions are expected to activate different prefrontal areas for Novel followed by the Goal (N.G), simultaneous Novel and Goal followed by the Goal (NG.G) and Zero followed by the Goal (Z.G), i.e. different prefrontal activations should be found in Z.G vs. G.G, N.G vs. G.G, NG.G vs. Z.G, NG.G vs. G.G, and N.G vs. Z.G contrasts.

H3: Auditory modelling may be better defined over motor control through modelling at multitask cognitive computation.

## Methods

### Participants

Twelve adults participated in the present study (mean age: 30.75 ± 8.8 years; range 18–48 years). All subjects self-reported normal hearing and no history of known neurological illness. The study was approved by the University of Dundee Institutional Review Board and NHS Tayside and was performed in accordance with the ethical standards for radiology intervention by NHS Tayside. All participants gave informed written consent before participating in the study. One healthy participant was excluded because the structural MRI was lost, leaving 11 healthy (10 right-handed) subjects.

### Experimental design

Subjects were asked to perform an odd/even auditory number decision task during simultaneous scalp EEG and fMRI recordings. The paradigm was composed of 400 trials, with trials chosen pseudo-randomly from one of four different conditions. Each trial consisted of a sound stimulus. The parameters of the stimuli are given in Table 1. Participants were asked to respond by pressing a button as quickly as possible without sacrificing accuracy. Participants used the index and middle fingers of their right hand. The Inter-Trial Interval (ITI) was between 1900 and 2100 ms. The task was presented in one single block (400 trials) with each of the four conditions presented in random order. Stimulus sequence was the same across all participants.

**Table 1:** Stimuli combinations for the simultaneous EEG/fMRI experiment

Stimuli name	Number of presentations	Code processed	Stimuli	
			S2	
			Type	Time
Standard goal stimuli	250	G	Number	300 ms
Non-goal stimuli	50	Z	Zero	200 ms
Simultaneous novel and goal	50	NG	Number + Novel	300 ms
Novel stimuli	50	N	Novel	55, 135, 200 ms

*SOA* stimulus-onset asynchrony

### Stimuli

Stimuli were sounds presented using Nordic Neurolab Electrostatic Headphones at 80 dB sound pressure level. Sound files were stereo with 16-bit resolution and 22,050 Hz sampling rate.

In the standard goal stimulus condition (G), the stimulus (S2) was a number of 300 ms duration. In the non-goal stimulus condition (Z), S2 was the number zero of 300 ms duration. In the novel only condition (N), S2 was a novel sound of 55, 135 or 200 ms duration. Finally, in the simultaneous novel and goal condition (NG), S2 was a number of 300 ms duration simultaneously presented with a lateralized novel sound of 100 ms duration.

### EEG recording

EEG data were recorded continuously using a 64-channel EEG acquisition system designed especially for the MR environment (Vision Recorder, Brain Product, Inc., Munich, Germany). The electrode placement followed the extended international 10–20 system, using FCz as a reference electrode. Amplified signals were digitized at 5000 Hz with a 16-bit resolution. All electrode impedances were < 20 kΩ. Data were band-pass filtered between 0.016–250 Hz during data acquisition. Trials with excessive peak-to-peak deflections, amplifier clipping or excessive high-frequency (EMG) activity were excluded before analysis. This data has provided P300 results across averaging participants, but noise data was not able to combine results with fMRI acquisition.

### fMRI acquisition and analysis

Whole-brain images (30 slices; 2.6 mm thick, 0.4 mm gap, 64 × 64 pixels in-plane resolution, overall resolution 3.75 × 3.75 × 5 mm) were collected on a 3-T Trio Siemens scanner using an echo-planar imaging sequence. Scans were acquired with a repetition time of 2.5 s and echo time of 30 ms. Additionally, a T1-weighted structural scan was acquired for each subject (1 mm isotropic resolution). SPM8 was used for both pre-processing and statistical analysis [21]. Images were spatially realigned to reduce movement artefacts. Mean image and structural data were used for co-registration, and co-registration results were then used to produce normalized images. Images were spatially normalized to the MNI template and spatially smoothed using a Gaussian kernel of 8 mm full-width at half height. The BOLD signal was then high-pass filtered with a cut-off of 256 s.

**Fig. 1:**
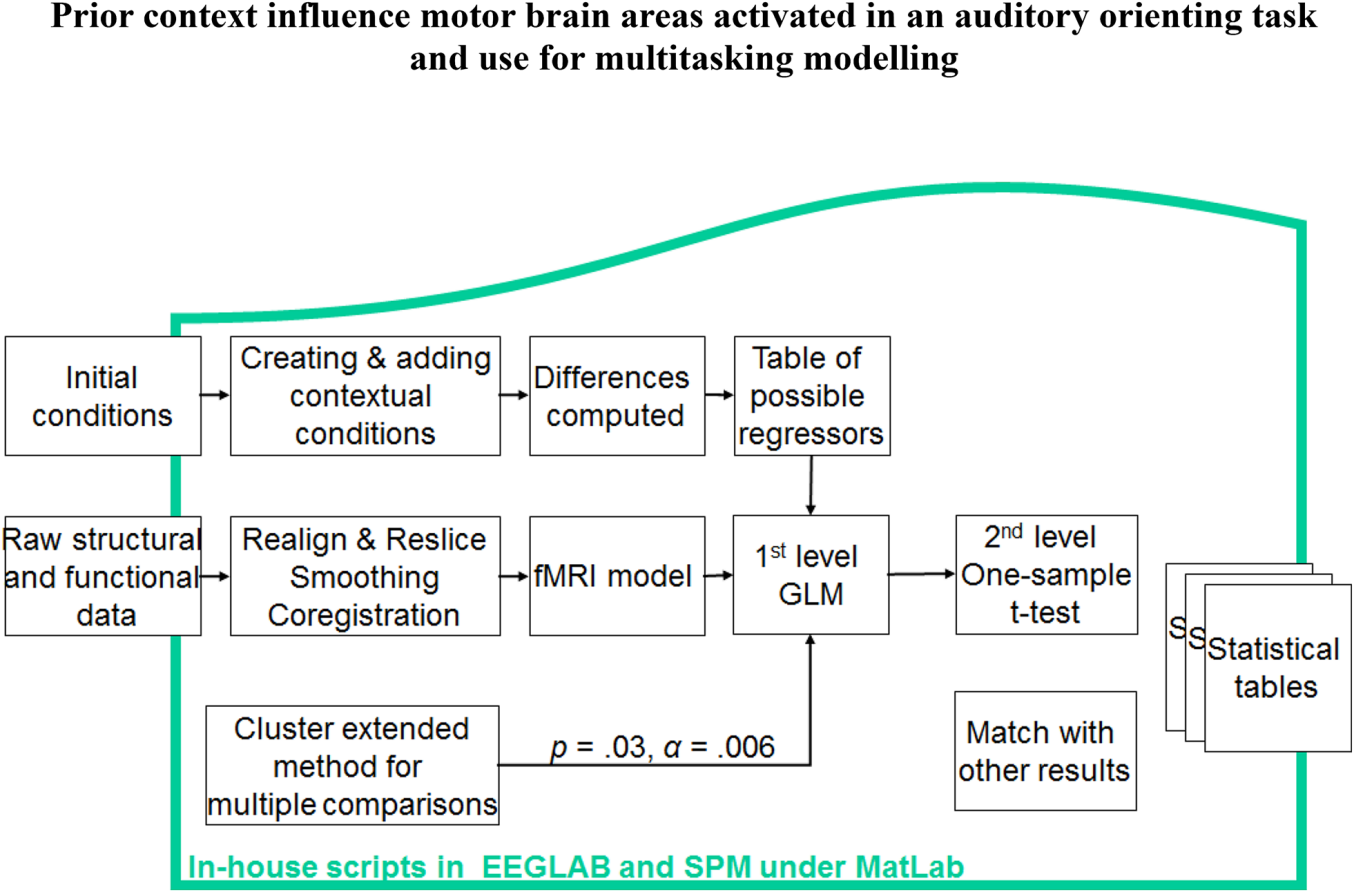
Preprocessing and analysis diagram used for the auditory oddball task in the simultaneous EEG and fMRI recording

A subset of different possible regressors was used: (1) from initial conditions; (2) extended contextual conditions (see Fig. 1). To explore the main effects of conditions and contextual analysis in the whole group, we adopted a voxel-wise type I error threshold of *α* = 0.03 and used the cluster extent method to correct for multiple comparisons [22]. Areas exceeding a corrected cluster-wise type I error threshold of *α* = 0.006 (*k* > 1055 voxels, equivalent in spatial extent to 15 original non-resampled voxels) were selected for further analysis to determine the directionality of category-specific main effects and to test for interactions. Given that the cluster extent method is not as stringent as false discovery rate (FDR) or family wise error (FWE), we have chosen *α* = 0.03. With these 1055 voxels, the second-level random effects analyses were conducted. FDR script (https://warwick.ac.uk/fac/sci/statistics/staff/academic-research/nichols/software/fdr/fdrm) was conducted on SPM, employing Nichols later Matlab script [23]. These analyses were achieved by entering the six covariate images of interest into one-group t-test. Due to the small number of participants for orienting (*n* = 6) and non-orienting (*n* = 5), only statistical analysis within ‘distracted’ participants (*n* = 6) and the whole (*n* = 11) groups was carried out.

### Synthesis for auditory and motor modelling (conclusions)

Interpretation of results would allow to model the function of auditory and motor function in an auditory oddball task. Therefore, results would allow having a better grasp of motor and auditory interaction on Goal-Driven tasks.

## Results

### Behavioural results

Both accuracy and mean response latencies were examined in the critical trials common to our two goal stimulus conditions, Goal (G) and the simultaneous Novel and Goal (NG). Overall, participants performed well (94% accuracy of goal trials). The proportion of correct responses was analysed using a 2-way ANOVA. The main effect of condition was not significant across subjects (*F*(1,11) = 0.43, *p* = 0.5136).

**Fig. 2:**
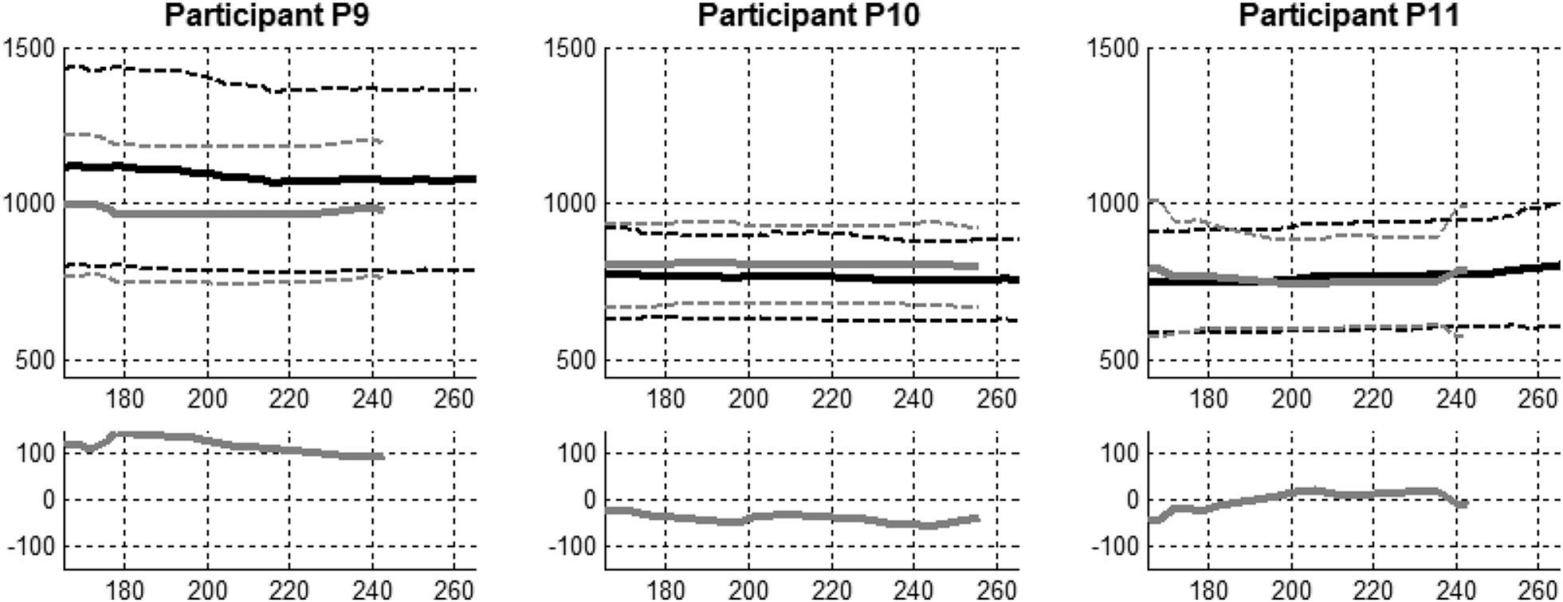
Running average of RT for conditions G (coloured in black) and NG (coloured in gray) in the 12 participants. Solid lines in the upper plots are the means at every condition (black for standard target condition and gray for noisy target). In the bottom plots the difference of the RTs between G and NG is shown

A time series analysis using a running average of reaction times was conducted in each participant to explore the basis of these non-significant results and the small effect size (< 0.01). Running average reaction times in the 12 control participants for conditions G (coloured in black) and NG (coloured in gray) are illustrated in Fig. 2.

Solid lines in the upper plots are the means for every condition (black for standard Goal stimuli, gray for the simultaneous Novel and Goal). In the bottom plots the difference of the RTs between the G condition minus the NG condition are shown. There the average and standard deviation calculation of reaction times was run, taking as the centre, the central trial plus and minus 75 trials (condition G) or 15 trials (condition NG) across the whole of the possible accurately answered trials (this explains why the measure does not start from 0 and finish at 400) rendering 151 trials (condition G) and 31 trials (condition NG). This is called running average of Reaction time or running average RT.

Novel distractors slowed RTs in 6 participants (7, 8, 10, 14, 15 and 16), speeded up reaction times in 4 participants (4, 5, 9 and 12) while 2 participants (6 and 11) showed no differences. In Fig. 2 the running average RTs for the G and NG conditions are illustrated along with the average difference between the two conditions.

Overall, the lack of significant differences in RT in the two-way ANOVA may be explained by the individual differences in pattern of the running average reaction times in the different conditions. Some individuals clearly show distraction effects while others do not.

### fMRI results based on the immediately preceding context analysis included in the analysis for ‘distracted’ participants

Continuing with the focus of the condition of the trial immediately prior to the current trial as suggested in controls and schizophrenic participants [3], the classical fMRI analysis was extended. The contextual cases tested in this fMRI analysis were: Z.G vs. G.G, N.G vs. G.G, NG.G vs. Z.G, NG.G vs. G.G, and N.G vs. Z.G.

Common different brain area activations are in the Left Parietal Precuneus, the Right Sub lobar Insula and in the Right Temporal Lobe in the Superior Temporal Gyrus (R STG). In the last case, L STG has different brain activation except for the N.G vs. Z.G contrast (this is discussed in Sect. 4.2).

**Fig. 3:**
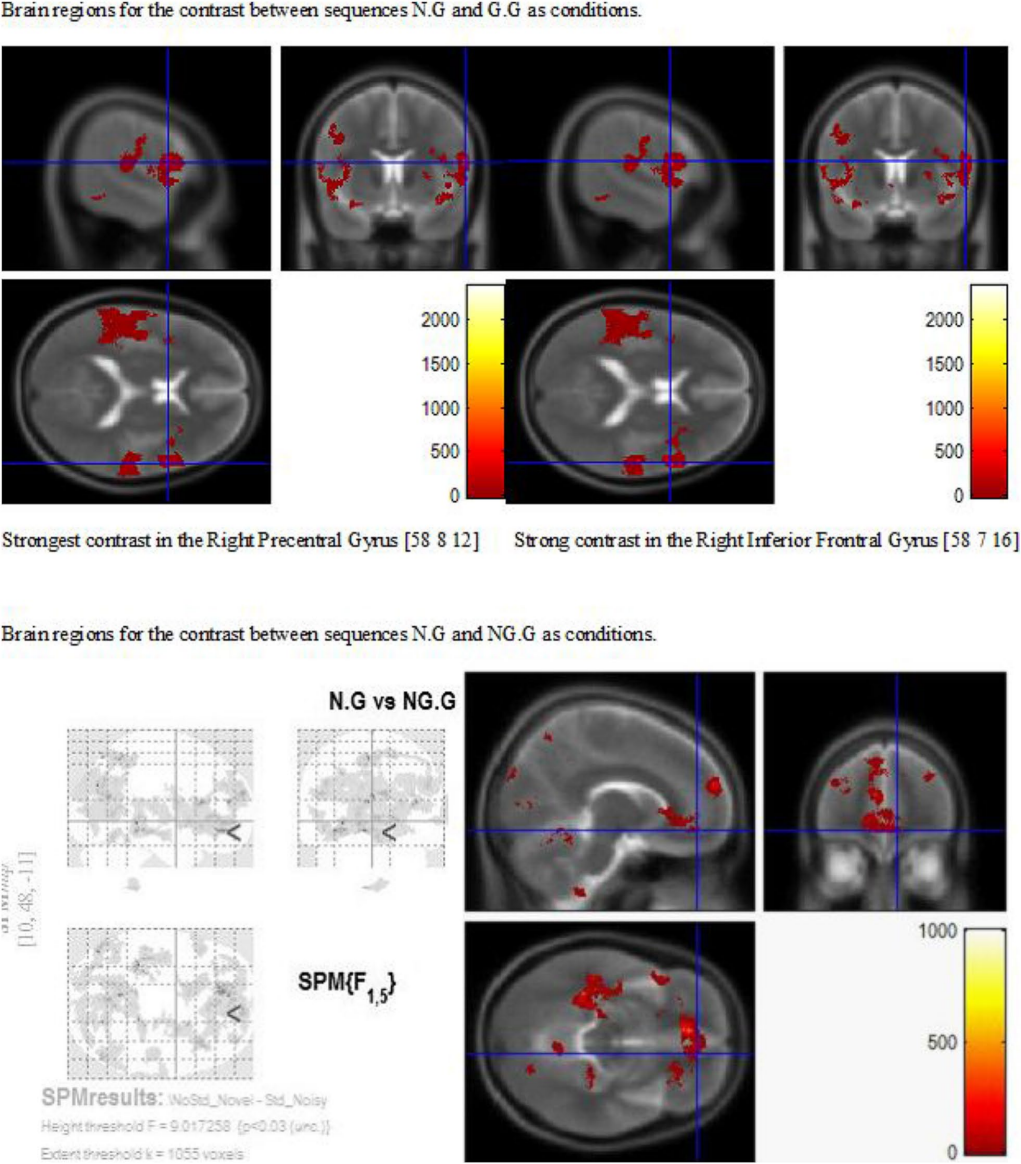
Cross-sectional images with the blue cross bars point to the maximum F value in brain regions for the contrast between sequences N.G and G.G as conditions on the top and sequences N.G and NG.G as conditions on the bottom

Table 2 lists the differences observed in the contrast between Z.G and G.G. Both hemispheres in frontal, temporal, parietal, occipital and limbic brain areas showed differences strongly biased to the Z.G contextual condition. According to the results, there are no brain areas with the same BA in the positive and negative contrasts, and only the Left Medial Frontal Gyrus with different Brodmann Areas (BA), BA 6 biased to Z.G and the BA 9 biased to the G.G condition. The left and right frontal areas in Inferior and Middle Frontal Gyrus (IFG and MFG) are positive activated. Also, positive differences were found for R MFG, R IFG, and R IPL, and L IPs and R IPs (Fig. 3).

**Table 2:** Brain areas and statistical results of Orienting Group (*n* = 6) with *p* < .03 (uncorrected) and 1055 voxels activated for zero followed by the goal and goal followed by the goal conditions: Z.G vs G.G

	Significant brain areas activated	Voxels with maximum T value					*Brodmann areas*		Significant brain areas activated	Voxels with maximum T value					*Brodmann *areas
	Positive difference	Positive Statistics		Coordinates					Negative difference	Negative Statistics		Coordinates			
		*T* value	*p* value	*x*	*y*	*z*				*T* value	*p* value	x	y	z	
*Frontal lobes*								*Frontal lobes*							
**1**	**L MedialFrontal Gyrus**	5.26	.002	− 1	− 24	59	6	**1**	**L MedialFrontal Gyrus**	4.47	0.0033	− 12	39	23	
2	L ParacentralLobule	2.71	.021	− 2	− 29	59	6								
3	R InferiorFrontal Gyrus	14.80	< .001	23	28	− 10	47								
4	R MedialFrontal Gyrus	3.66	.007	4	− 26	58	6								
5	R MiddleFrontal Gyrus	22.96	< .001	24	26	− 11	11								
6	R ParacentralLobule	5.76	.001	10	− 29	57	3,31,5,6								
7	R PostcentralGyrus	3.88	.006	17	− 34	61	4								
8	R PrecentralGyrus	4.83	.002	18	− 15	66	4,6								
9	R SuperiorFrontal Gyrus	5.36	.002	28	− 7	66	6								
*Parietal lobes*								*Parietal lobes*							
10	L PostcentralGyrus	3.22	.012	− 57	− 27	19	40								
11	L Precuneus	7.89	< .001	− 18	− 73	23	31								
12	R AngularGyrus	7.43	< .001	50	− 68	34	39								
13	R InferiorParietal Lobule	7.42	< .001	54	− 58	40	39,40								
14	R PostcentralGyrus	3.76	.007	13	− 34	63	3								
15	R Precuneus	4.12	.005	44	− 71	41	19,39								
*Temporal lobes*								*Temporal lobes*							
16	L MiddleTemporal Gyrus	7.71	< .001	− 60	− 30	2	19,21,22,39								
17	L SuperiorTemporal Gyrus	10.07	< .001	− 50	− 31	3	21,22,41,42								
18	L TransverseTemporal Gyrus	5.80	.001	− 41	− 32	11	41								
19	R MiddleTemporal Gyrus	3.83	.006	46	− 63	27	39								
20	R SuperiorTemporal Gyrus	5.86	.001	38	− 55	17	13,22,41,42								
21	R TransverseTemporal Gyrus	3.49	.009	51	− 24	10	41								
*Occipital lobes*															
22	L Cuneus	15.58	< .001	− 15	− 79	27	18,19,7	2	R FusiformGyrus	5.35	0.0015	28	− 48	− 7	37
23	L Precuneus	9.50	< .001	− 19	− 77	28	31	3	R ParahippocampalGyrus	4.96	0.0021	26	− 48	− 9	37
24	L SuperiorOccipital Gyrus	5.27	.002	− 35	− 83	29	19								
25	R MiddleTemporal Gyrus	7.69	< .001	39	− 58	16	19								
26	R Precuneus	3.97	.005	14	− 72	26	31								
*Limbic lobes*															
27	L CingulateGyrus	6.94	< .001	− 10	− 55	28	31	4	L AnteriorCingulate	3.38	0.0098	− 12	36	24	32
28	L PosteriorCingulate	11.93	< .001	− 6	− 57	25	31	5	R ParahippocampalGyrus	3.80	0.0063	31	− 47	− 6	19
29	L Precuneus	4.98	.002	− 12	− 43	37	31								
*Deep gray (Sub lobar areas)*								*Deep gray (Sub lobar areas)*							
30	L LentiformNucleus	3.04	.014	− 15	− 5	4		6	R ThalamusPulvinar	6.94	0.0005	9	− 30	9	
31	L LentiformNucleusLateralGlobus Pallidus	4.02	.005	− 14	0	1									
32	L LentiformNucleusMedialGlobus Pallidus	3.59	.008	− 11	0	− 1									
33	L LentiformNucleusPutamen	3.36	.01	− 18	1	3									
34	L Thalamus	6.25	< .001	− 9	− 9	1									
35	L ThalamusMammillary Body	3.16	.012	− 13	− 20	2									
36	L ThalamusMedial DorsalNucleus	4.15	.004	− 7	− 20	3									
37	L ThalamusVentral AnteriorNucleus	3.19	.012	− 16	− 8	12									
38	L ThalamusVentral LateralNucleus	6.74	< .001	− 10	− 9	3									
39	L ThalamusVentral PosteriorLateral Nucleus	2.96	.016	− 17	− 22	1									
40	L ThalamusVentral PosteriorMedial Nucleus	3.24	.011	− 14	− 21	0									
41	R Claustrum	6.28	< .001	36	− 19	4									
42	R Insula	5.14	.002	41	− 18	4	13								
43	R LentiformNucleusPutamen	4.37	.004	24	1	14									
44	R Thalamus	19.51	< .001	10	− 24	0									
	*Additional regions*							*Additional regions*							
45	L MidbrainSubstania Nigra	5.64	.001	− 14	− 21	− 6		7	L Anterior LobeCulmen	2.94	0.016	− 1	− 46	− 6	
46	R MidbrainRed Nucleus	3.58	.008	8	− 20	− 6		8	R Anterior LobeCulmen	3.65	0.0074	2	− 44	− 5	
47	R MidbrainSubstania Nigra	4.06	.005	11	− 22	− 7									

Anatomical labels and associated *T* statistical values are listed. *t* scores from the omnibus analyses of 6 participants for each ROI are presented

**Fig. 4:**
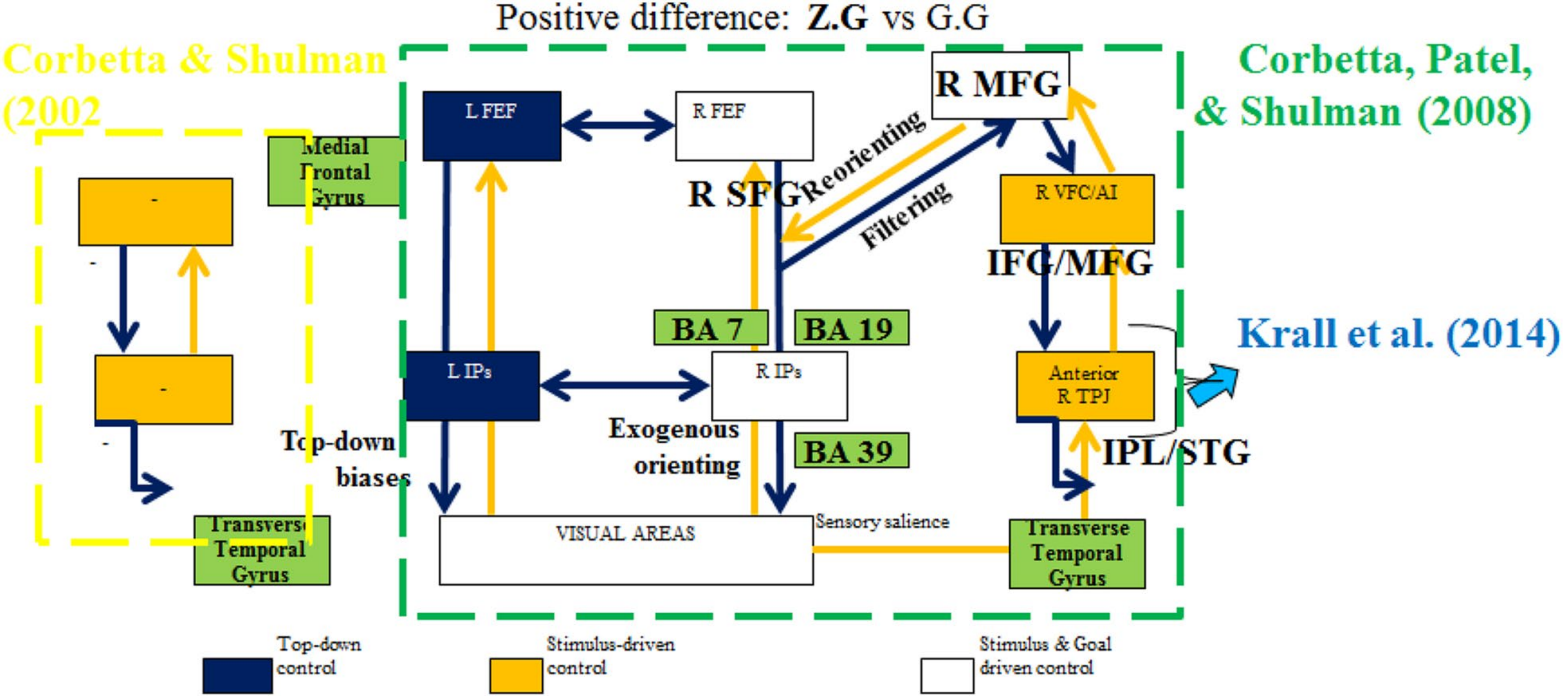
Comparison of the positive and negative difference of the brain areas for the contrast Z.G vs. G.G, showing an interaction between Filtering and Reorienting mode of attention

Table 3 lists the differences observed in the contrast of sequences N.G and G.G. Both hemispheres in frontal, temporal, parietal and right limbic brain areas showed differences strongly biased to the N.G contextual condition. According to the results, there are no common areas for positive and negative contrast. There are strong frontal differences in R Precentral Gyrus and the R IFG and in 5 other frontal areas. Results showed that the greatest differences measured occurred towards the most frontal area of the brain, with the greatest frontal differences measuring up to 37 mm in the left MFG and up to 28 mm in the right MFG, which means that frontal activation is larger in the left hemisphere when the Novel is presented immediately before the present Goal stimulus. This left lateralization response is consistent with the present Goal stimulus. Figure 4 shows this contrast.

**Table 3:** Brain areas and statistical results of Orienting Group (*n* = 6) with *p*< .03 and 1055 voxels activated for novel and goal stimulus vs goal and goal conditions: N.GvsG.G

	Significant brain areas activated	Voxels with maximum Z score					Brodmann areas
	Positive difference	Statistics		Coordinates			
		*T *value	*p *value	*x*	*y*	*z*	
*Frontal lobes*							
1	L InferiorFrontal Gyrus	14.38	< .001	− 42	13	− 7	47
2	L MiddleFrontal Gyrus	29.22	< .001	− 24	37	− 8	11,47,6
3	L PrecentralGyrus	14.09	< .001	− 39	− 18	41	4
4	L SubcallosalGyrus	4.95	.002	− 19	15	− 11	47
5	R CingulateGyrus	6.62	< .001	15	14	37	32
6	R InferiorFrontal Gyrus	37.42	< .001	57	8	14	44,47,6,9
7	R MedialFrontal Gyrus	3.26	.011	10	28	30	6,9
8	R MiddleFrontal Gyrus	16.43	< .001	53	6	34	6,8,9
9	R PrecentralGyrus	48.74	< .001	58	8	12	4,44,6
*Parietal lobes*							
10	L InferiorParietal Lobule	21.01	< .001	− 64	− 24	28	40
11	L PostcentralGyrus	35.14	< .001	− 61	− 21	28	2
12	L Precuneus	19.25	< .001	− 13	− 56	51	7
13	R InferiorParietal Lobule	9.72	< .001	66	− 34	30	40
14	R PostcentralGyrus	5.15	.002	57	− 22	43	2,3
15	R Precuneus	15.35	< .001	18	− 58	55	7
*Temporal lobes*							
16	L FusiformGyrus	18.68	< .001	− 33	− 41	− 16	20
17	L InferiorTemporal Gyrus	15.19	< .001	− 56	− 9	− 16	21
18	L Sub Gyral	15.58	< .001	− 39	− 12	− 8	21
19	L SuperiorTemporal Gyrus	20.46	< .001	− 48	6	− 3	22,38
20	R MiddleTemporal Gyrus	4.67	.003	59	− 60	11	37,39
21	R SuperiorTemporal Gyrus	7.05	< .001	64	− 40	21	0,0,13,22,42
*Limbic lobes*							
22	R CingulateGyrus	15.48	< .001	16	− 27	39	24,31,32,9
*Deep gray (Sub lobar areas)*							
23	L CaudateCaudate Head	2.46	.028	− 11	15	− 6	
24	L Insula	27.92	< .001	− 52	− 34	19	13
25	L LentiformNucleusMedialGlobus Pallidus	13.90	< .001	− 15	− 4	− 3	
26	L LentiformNucleusPutamen	3.49	.009	− 19	12	− 7	
27	R Insula	17.34	< .001	42	12	13	13
*Additional regions*							
28	L Anterior LobeCulmen	14.93	< .001	− 24	− 40	− 17	
29	R Anterior LobeCulmen	16.67	< .001	12	− 60	− 10	
30	R Anterior Lobe	3.19	.012	11	− 42	− 27	
31	R Posterior LobeCerebellarTonsil	3.93	.006	6	− 47	− 33	
32	R Posterior LobeDeclive	22.13	< .001	12	− 62	− 11	
	Positive difference						
*Limbic lobes*							
1	Right Limbic LobeAnteriorCingulate GM	4.23	.004	5	19	19	6, 33
*Additional regions*							
2	Left CaudateGMCaudate Body	7.89	< .001	− 9	17	13	2

Anatomical labels and associated *T* statistical values are listed. *T* scores from the omnibus analyses of 6 participants for each ROI are presented

Moreover, the PreCentral Gyrus is activated differently between this N.G and G.G contrast, with a clearly right lateralized bias. Bearing in mind that this area was not found in the results for the N and G contrast, thus the Novel before a Goal makes more contribution to different motor area activations. Therefore, this result suggests that attention to the task by the participants produces different motor control in N vs. G contrast and in N.G and G.G contrast. This is addressed in the discussion. Overall these differences in the Prefrontal Cortex by the trial before the G condition in analysis support hypothesis H2.

Table 4 lists the observed differences for the contrast of sequences N.G and NG.G, showing frontal differences in 10 regions. Both hemispheres in frontal, temporal, parietal and limbic brain areas showed differences strongly biased to the N.G contextual condition. According to the results, the Right IFG with BA 13, Right SFG with BA 6 and the Right Cingulate Gyrus with BA 24 are activated with both positive and negative contrast (see the highlighted results in Table 7). In addition, the left Precentral Gyrus is activated differently in this contrast, which informs different motor response than the other contrasts. Again, there are frontal differences in left and right MFG (up to 46 mm and 44 mm, respectively). Results showed that the greatest differences measured occurred towards the most frontal area of the brain, with the greatest frontal differences measuring up to 50 mm in the left SFG and up to 56 mm in the right SFG, having the more frontal activation in the right hemisphere. Overall these differences in the Prefrontal Cortex by the trial before the G condition in analysis are supporting hypothesis H2 and suggest the more frontal activation for the switching from simultaneous Novel and Goal to the Goal which is also concordant with Koechlin’s model (2003) of the frontal episodic attention control and with Corbetta’s model [20] lateralizing to the right hemisphere. Figure 4 on the bottom shows this contrast. Our results also extended the idea on steady-state visual evoked potentials (SSVEP) where frontal electrodes in 2-oddball attention tasks were found responsible for suppression of distractor responses [24], i.e. how different 2-oddball task maybe seen locally in prior context in the present 4-oddball task experiment.

**Table 4:** Brain areas and statistical results of the 'distracted' subgroup (*n* = 6) with *p*< .03 and 1055 voxels activated for novel and goal stimulus vs simultaneous novel and goal and goal conditions -N.GvsNG.G

	Significant brain areas activated	Voxels with maximum T value						Brodmann areas		Significant brain areas activated	Voxels with maximum T value						Brodmann areas
		Statistics			Coordinates						Statistics			Coordinates			
		*F *value	*Z *score	*p *value	*x*	*y*	*z*				*F *value	*Z *score	*p *value	*x*	*y*	*z*	
*Frontal lobes*									*Frontal lobes*								
'1'	R InferiorFrontal Gyrus '	'6.8288'	'3.2831'	'0.00051345'	'43'	'18'	'13'	13,45	'1'	L InferiorFrontal Gyrus '	'11.3373'	'3.9072'	'4.6685e−005'	'− 46'	'19'	'− 7'	[4,45,47
'2'	R MedialFrontal Gyrus '	'13.2842'	'4.0896'	'2.1606e−005'	'13'	'10'	'44'	32,6	'2'	L MedialFrontal Gyrus '	'24.6969'	'4.7504'	'1.015e−006'	'− 5'	'46'	'12'	10,11,9
'3'	R MiddleFrontal Gyrus '	'9.2042'	'3.6581'	'0.00012703'	'19'	'− 10'	'60'	6	'3'	L MiddleFrontal Gyrus '	'14.9533'	'4.222'	'1.2106e−005'	'− 26'	'34'	'− 14'	11,6
'4'	R PrecentralGyrus '	'2.6202'	'1.9905'	'0.023268'	'11'	'− 17'	'63'	6	'4'	L ParacentralLobule '	'15.6203'	'4.2701'	'9.7711e−006'	'− 9'	'− 39'	'51'	5
'5'	R Sub Gyral'	'3.9077'	'2.5336'	'0.0056444'	'18'	'− 6'	'56'	6	'5'	L PrecentralGyrus '	'6.8314'	'3.2835'	'0.00051254'	'− 57'	'14'	'6'	44
'6'	R SuperiorFrontal Gyrus '	'6.3615'	'3.1909'	'0.00070909'	'9'	'− 15'	'67'	6	'6'	L SuperiorFrontal Gyrus '	'10.9159'	'3.8627'	'5.6065e−005'	'− 10'	'50'	'23'	6,8,9
									'7'	R InferiorFrontal Gyrus '	'11.9146'	'3.965'	'3.6697e−005'	'34'	'16'	'− 15'	13,47
									'8'	R MedialFrontal Gyrus '	'11.606'	'3.9345'	'4.1678e−005'	'5'	'44'	'− 12'	10,11
									'9'	R ParacentralLobule '	'10.0156'	'3.7604'	'8.4833e−005'	'2'	'− 35'	'53'	5.00
									'10'	R SuperiorFrontal Gyrus '	'10.5991'	'3.8279'	'6.4619e−005'	'35'	'56'	'17'	10,6,8
*Parietal lobes*									*Parietal lobes*								
'7'	R InferiorParietal Lobule '	'3.7673'	'2.4833'	'0.006508'	'46'	'− 28'	'30'	40	'11'	L AngularGyrus '	'11.6574'	'3.9397'	'4.0795e−005'	'− 46'	'− 70'	'36'	39
'8'	R PostcentralGyrus '	'4.0264'	'2.5747'	'0.0050157'	'66'	'− 17'	'19'	40,43	'12'	L Precuneus'	'31.6291'	'4.9934'	'2.9662e−007'	'− 5'	'− 49'	'52'	19,31,39,7
									'13'	R AngularGyrus '	'12.6358'	'4.0326'	'2.7577e−005'	'50'	'− 67'	'31'	39
									'14'	R Precuneus'	'13.6899'	'4.1236'	'1.8653e−005'	'4'	'− 54'	'53'	7
									'15'	R SuperiorParietal Lobule '	'10.0724'	'3.7671'	'8.2563e−005'	'16'	'− 63'	'55'	7
*Temporal lobes*									*Temporal lobes*								
'9'	R MiddleTemporal Gyrus '	'3.6098'	'2.4246'	'0.007662'	'65'	'− 9'	'-3'	21.00	'16'	L AngularGyrus '	'8.4922'	'3.5591'	'0.00018608'	'− 48'	'− 73'	'30'	39
'10'	R SuperiorTemporal Gyrus '	'8.1246'	'3.504'	'0.00022918'	'68'	'− 31'	'14'	22,41,42	'17'	L FusiformGyrus '	'4.578'	'2.7506'	'0.0029747'	'− 36'	'− 38'	'− 11'	37
'11'	R TransverseTemporal Gyrus '	'6.3783'	'3.1944'	'0.00070067'	'49'	'− 20'	'12'	41,42	'18'	L MiddleTemporal Gyrus '	'11.4275'	'3.9165'	'4.4928e−005'	'− 50'	'− 62'	'22'	39
									'19'	L Sub Gyral'	'7.5006'	'3.4032'	'0.00033296'	'− 39'	'− 11'	'− 10'	21
									'20'	L Sub GyralHippocampus'	'28.6257'	'4.8967'	'4.8734e−007'	'− 29'	'− 38'	'− 1'	
									'21'	L SuperiorTemporal Gyrus '	'8.9854'	'3.6287'	'0.00014243'	'− 51'	'− 61'	'21'	38,39
									'22'	R Sub GyralHippocampus'	'6.3239'	'3.1832'	'0.00072835'	'29'	'− 30'	'− 6'	
										*Occipital lobes*							
									'23'	L Cuneus'	'8.4635'	'3.5549'	'0.00018907'	'− 20'	'− 67'	'17'	18.00
									'24'	L Precuneus'	'15.5131'	'4.2625'	'1.0107e−005'	'− 3'	'− 59'	'20'	23,31
									'25'	R Cuneus'	'8.2024'	'3.5159'	'0.00021915'	'21'	'− 81'	'12'	17.00
'12'	R CingulateGyrus '	'18.8987'	'4.475'	'3.821e− 006'	'14'	'7'	'43'	24,32	'26'	L AnteriorCingulate '	'18.0094'	'4.4239'	'4.8477e−006'	'− 10'	'34'	'− 10'	32.00
									'27'	L CingulateGyrus '	'23.9905'	'4.7212'	'1.1723e−006'	'− 5'	'22'	'27'	24,32
									'28'	L ParahippocampalGyrus '	'18.2909'	'4.4404'	'4.4905e−006'	'− 30'	'− 37'	'− 7'	27,28,36,37
									'29'	L PosteriorCingulate '	'9.8112'	'3.7356'	'9.3642e−005'	'− 16'	'− 56'	'7'	30.00
									'30'	R AnteriorCingulate '	'14.6069'	'4.1961'	'1.358e−005'	'3'	'30'	'0'	24.00
									'31'	R CingulateGyrus '	'2.8155'	'2.0862'	'0.01848'	'4'	'− 8'	'34'	24.00
									'32'	R ParahippocampalGyrus '	'3.8807'	'2.5241'	'0.0058'	'20'	'− 34'	'− 9'	27,30,35,36
									'33'	R ParahippocampalGyrus Hippocampus'	'5.0219'	'2.8762'	'0.0020122'	'29'	'− 32'	'− 5'	
'13'	R Claustrum'	'5.8839'	'3.0882'	'0.0010067'	'30'	'4'	'15'		'34'	L CaudateCaudate Head'	'10.8174'	'3.852'	'5.8572e−005'	'− 8'	'10'	'− 4'	
'14'	R Insula'	'17.8313'	'4.4133'	'5.0914e−006'	'37'	'19'	'10'	13	'35'	L LentiformNucleusLateralGlobus Pallidus'	'11.4007'	'3.9137'	'4.5442e−005'	'− 13'	'6'	'− 6'	
'15'	R LentiformNucleusMedialGlobus Pallidus'	'16.9369'	'4.358'	'6.5617e−006'	'18'	'− 9'	'1'		'36'	R CaudateCaudate Tail'	'2.6688'	'2.0148'	'0.02196'	'28'	'− 39'	'7'	
'16'	R LentiformNucleusPutamen'	'21.3899'	'4.6042'	'2.0707e−006'	'22'	'9'	'0'		'37'	R Thalamus'	'14.8709'	'4.2159'	'1.2439e−005'	'22'	'− 30'	'2'	
'17'	R Thalamus'	'5.7299'	'3.0531'	'0.0011326'	'14'	'− 13'	'0'		'38'	R ThalamusPulvinar'	'9.7345'	'3.7261'	'9.7229e−005'	'22'	'− 33'	'3'	
'18'	R ThalamusVentral PosteriorLateral Nucleus'	'7.1802'	'3.3476'	'0.00040761'	'21'	'− 21'	'8'		'39'	'L Anterior LobeCulmen'	'4.9283'	'2.8508'	'0.0021805'	'− 13'	'− 33'	'− 11'	
'19'	'L Anterior LobeCulmen'	'7.2062'	'3.3522'	'0.00040084'	'0'	'− 59'	'− 10'		'40'	'R Anterior LobeCulmen'	'6.581'	'3.2351'	'0.00060789'	'13'	'− 39'	'− 12'	
'20'	'R Anterior LobeCerebellarLingual '	'6.4307'	'3.205'	'0.0006752'	'10'	'− 47'	'− 13'										
'21'	'R Anterior LobeCulmen'	'11.0456'	'3.8766'	'5.2955e−005'	'6'	'− 57'	'− 10'										
'22'	'R Anterior LobeFastigium'	'3.3541'	'2.3238'	'0.010068'	'6'	'− 50'	'− 19'										
'23'	'R Anterior Lobe'	'4.249'	'2.6486'	'0.0040416'	'23'	'− 48'	'− 25'										
'24'	'R Posterior LobeCerebellarTonsil '	'6.4246'	'3.2038'	'0.00067809'	'27'	'− 58'	'− 33'										
'25'	'R Posterior LobeDeclive'	'15.3791'	'4.253'	'1.0547e−005'	'4'	'− 58'	'− 11'										
'26'	'R Posterior LobePyramis'	'3.6933'	'2.456'	'0.007024'	'10'	'− 69'	'− 23'										

Anatomical labels and associated *T* statistical values are listed. *T* scores from the omnibus analyses of 6 participants for each ROI are presented

Table 4 also shows the frontal differences in the left and right Anterior Cingulate Cortex (ACC, up to 34 mm and 30 mm, respectively), this is consistent with the view that ACC is involved in conflict monitoring (reviewed by van Veen and Carter [25]) which is the previous context in our analysis.

In Table 5, the contrast of sequences N.G and Z.G is shown. Both hemispheres in occipital and limbic brain areas showed differences strongly biased to the Z.G contextual condition and both hemispheres showed activation for frontal, temporal and parietal in positive and negative contrasts. According to the results, the Left MedialFrontal Gyrus, Left SFG, Right MedialFrontal Gyrus, Right MFG, Right Precentral Gyrus, Right SFG, Left MiddleTemporal Gyrus and Right STG with different BAs are activated with both positive and negative contrast (see the highlighted results in Table 5). Also, Table 5 showed differences in several frontal regions biased to the N.G condition. Again, there are frontal differences in the left and right MFG (up to 46 mm and 44 mm, respectively). Results showed that the greatest differences measured occurred towards the most frontal area of the brain, with the greatest frontal differences measuring up to 50 mm in the left SFG and up to 56 mm in the right SFG, having more frontal activation in the right hemisphere. Overall these differences in the Prefrontal Cortex by the trial before the G condition in analysis support hypothesis H2 and suggest more frontal activation for the switching from simultaneous Novel and Goal to the Goal which is also concordant with Koechlin’s model of the frontal episodic attention control and with Corbetta’s model lateralizing to the right hemisphere [20].

**Table 5:** Brain areas and statistical results of the 'distracted' subgroup (*n* = 6) with *p* < .03 and 1055 voxels activated for simultaneous novel and goal and goal stimulus vs zero and goal stimulus conditions—NG.G vs Z.G

	Significant brain areas activated	Voxels with maximum *T*-value					Brodmann areas	Significant brain areas activated	Voxels with maximum *T*-value					Brodmann areas
	Positive difference	Statistics		Coordinates				Positive difference	Statistics		Coordinates			
		*T*	*p*	*x*	*y*	*z*			*T*	*p*	*x*	*y*	*z*	
*Frontal lobes*								*Frontal lobes*						
1	**L MedialFrontal Gyrus**	4.46	0.003	− 5	48	26	10, 9	1 L InferiorFrontal Gyrus	8.50	< . 001	− 34	22	8	13
								2 **L MedialFrontal Gyrus**	20.68	< . 001	− 2	− 15	63	32.6
								3 L MiddleFrontal Gyrus	9.92	< . 001	− 25	− 10	56	6
								4 L ParacentralLobule	7.28	< . 001	− 13	− 32	51	5
								5 L PostcentralGynus	9.79	< . 001	− 19	− 28	57	4
								6 L PrecentralGyrus	12.61	< . 001	− 29	− 12	54	4, 43, 6
2	**L SuperiorFrontal Gyrus**	7.46	< .001	− 6	52	23	10, 9	7 **L SuperiorFrontal Gyrus**	21.05	< . 001	− 21	5	63	6
								8 R InferiorFrontal Gyrus	7.08	< . 001	50	40	− 13	47
3	**R MedialFrontal Gyrus**	6.49	< .001	7	61	18	10, 9	9 **R MedialFrontal Gyrus**	9.00	< . 001	3	− 17	56	6
4	**R MiddleFrontal Gyrus**	22.9	< .001	47	23	40	46, 8, 9	10 **R MiddleFrontal Gyrus**	13.40	< . 001	47	41	− 13	10, 11, 47
5	**R PrecentralGyrus**	4.93	0.002	41	17	37	9	11 **R PrecentralGyrus**	4.84	. 002	36	− 28	61	4
6	**R SuperiorFrontal Gyrus**	5.67	0.001	26	60	17	10	12 **R SuperiorFrontal Gyrus**	11.03	< . 001	4	6	51	6
*Parietal lobes*								*Parietal lobes*						
7	R AngularGyrus	9.05	< .001	49	− 66	30	39,40	13 L PostcentralGyrus	12.66	< . 001	− 18	− 31	58	3
8	R Precuneus	17.11	< .001	27	− 80	39	19, 39, 7	14 R InferiorParietal Lobule	9.12	< . 001	63	− 43	23	40
9	R SupramarginalGyrus Temporal lobes	11.63	< .001	61	− 51	34	40	15 R PostcentralGyrus Temporal lobes	7.38	< . 001	12	− 32	67	2, 3, 40, 5
*Temporal lobes*								*Temporal lobes*						
10	L FusiformGyrus	16.3	< .001	− 32	− 39	− 16	20, 37							
11	**L MiddleTemporal Gyrus**	3.64	0.007	− 37	− 81	18	19	16 **L MiddleTemporal Gyrus**	8.10	< . 001	− 64	− 19	− 3	21, 22, 39
12	L Sub Gyral	9.78	< .001	− 43	− 11	− 10	21	17 L SuperiorTemporal Gyrus	21.09	< . 001	− 65	− 31	14	22, 39, 41, 42
13	L Sub GyralHippocampus	11	0< .001	− 29	− 39	0		18 L TransverseTemporal Gyrus	12.15	< . 001	− 39	− 32	11	41
14	R CaudateCaudateTail	4.17	0.004	34	− 34	2								
15	R FusiformGyrus	4.48	0.003	44	− 64	− 14	37							
16	R MiddleTemporal Gyrus	10.59	< .001	38	− 60	28	19, 39							
17	R Sub GyralHippocampus	7.29	< .001	28	− 35	− 2		19 **R Superior Temporal Gyrus**	20.29	< . 001	37	− 33	17	22, 41, 42
18	**R SuperiorTemporal Gyrus**	4.97	0.002	56	− 63	27	39	20 R TransverseTemporal Gyrus	10.00	< . 001	43	− 30	11	41
*Occipital Lobes*								*Occipital Lobes*						
19	L FusiformGyrus	9.15	< .001	− 27	− 67	− 9	19							
20	L LingualGyrus	8.94	< .001	− 19	− 63	1	18, 19							
21	L MiddleOccipital Gyrus	9.05	< .001	− 35	− 84	10	18, 19							
22	L SuperiorOccipital Gyrus	2.71	0.021	− 36	− 82	23	19							
23	R Cuneus	11.24	< .001	19	− 84	36	17, 18, 19							
24	R FusiformGyrus	6.51	< .001	44	− 67	− 14	19							
25	R LingualGyrus	6.55	< .001	16	− 84	3	17, 18							
26	R MiddleOccipital Gyrus	8.13	< .001	38	− 83	18	18, 19							
27	R SuperiorOccipital Gyrus	3.04	0.014	33	− 80	33	19							
*Limbic lobes*								*Limbic lobes*						
28	L AnteriorCingulate	5.59	< .001	− 10	33	− 10	32							
29	L LingualGyrus	8.32	< .001	− 12	− 47	2	2							
30	L ParahippocampalGyrus	17.29	< .001	− 30	− 37	− 9	30,36,37							
31	L PosteriorCingulate	8.35	0.0002	− 21	− 67	6	30							
32	R AnteriorCingulate	8.16	< .001	4	36	− 4	24,32							
33	R ParahippocampalGyrus	16.37	< .001	22	− 33	− 1	19,27,28,30							
34	R ParahippocampalGyrus Amygdala	4.15	< .001	24	− 8	− 11								
	*Deep gray (Sub lobar areas)*							*Deep gray (Sub lobar areas)*						
35	L CaudateCaudate Body	3.99	.005	− 13	8	21		21 L Claustrum	7.10	< .001	− 32	− 22	9	
36	R CaudateCaudate Body	5.45	.001	14	14	17		22 L Insula	33.52	< .001	− 55	− 36	19	13
37	R ThalamusPulvinar	10.95	< .001	11	− 32	9	9	23 L LentiformNucleus	8.63	< .001	− 13	− 1	5	
38	R Amygdala	3.67	.007	23	− 10	− 10		24 L LentiformNucleusLateralGlobus Pallidu	11.54	< .001	− 13	1	4	
								25 L LentiformNucleusPutamen	11.33	< .001	− 15	3	3	
								26 L Thalamus	7.09	< .001	− 13	− 17	4	
								27 L ThalamusMammillary Body	5.10	.002	− 11	− 18	4	
								28 L ThalamusMedial DorsalNucleus	4.16	.004	− 8	− 15	6	
								29 L ThalamusVentral AnteriorNucleus	4.03	.005	− 16	− 8	11	
								30 L ThalamusVentral LateralNucleus	5.28	.002	− 10	− 12	3	
								31 L ThalamusVentral PosteriorLateral Nuclo	8.75	< .001	− 18	− 19	6	
								32 L ThalamusVentral PosteriorMedial Nuclo	6.03	< .001	− 16	− 20	6	
								33 R Claustrum	12.62	< .001	32	− 13	12	
								34 R Insula	27.58	< .001	39	− 27	17	13
								35 R LentiformNucleusPutamen	10.95	< .001	22	11	6	
								36 R Thalamus	38.66	< .001	10	− 12	1	
								37 R ThalamusPulvinar	8.62	< .001	20	− 23	15	
								38 R ThalamusVentral LateralNucleus Additional regions	13.42	< .001	10	− 14	3	
*Additional regions*								*Additional regions*						
								39 L MidbrainSubthalamic Nucleus	4.58	.003	− 11	− 11	− 2	
								40 R MidbrainSubthalamic Nucleus	23.56	< .001	12	− 10	− 1	
39	L Anterior LobeCulmen	7.42	< .001	− 33	− 60		− 24							
40	R Anterior LobeCulmen	9.02	< .001	8	− 45		1							
41	R Anterior LobeCulmenofVermis	8.98	< .001	3	− 59		1							
42	L Posterior LobeDeclive	13.74	< .001	− 37	− 72		− 17							
43	L Posterior LobeTuber	9.11	< .001	− 42	− 65		− 23							
44	R Posterior LobeDeclive	14.84	< .001	45	− 69		− 18							
45	R Posterior LobeTuber	13.98	< .001	40	− 72		− 24							
46	L MidbrainSubstania Nigra	6.79	< .001	− 9	− 15		− 10							
47	R MidbrainMammillary Body	5.07	0.002	4	− 10		− 8							
48	R MidbrainSubstania Nigra	4.65	0.003	7	− 14		− 9							

Anatomical labels and associated *T* statistical values are listed. *t* scores from the omnibus analyses of 6 participants for each ROI are presented

In Table 4, there are also differences in the left and right Anterior Cingulate Cortex (ACC). This is consistent with the view of ACC in conflict monitoring [25] which is the previous context in our analysis.

Table 6 shows the contrast of sequences N.G and Z.G. Both hemispheres in parietal brain areas showed differences strongly biased to the Z.G contextual condition and both hemispheres showed activation for frontal, temporal, occipital and limbic in positive and negative contrasts. According to the results, the Right SuperiorTemporal Gyrus with the BA 22 with both positive and negative contrast (see the highlighted results in Table 6). Further, Table 6 showed frontal differences in two frontal regions biased to the N.G condition. In these contrasts, there are frontal differences in right MFG biased on N.G (up to 37 mm). The other great frontal difference is up to 32 mm in the right IFG. Therefore, the more frontal activation occurs in the left hemisphere. Overall, these differences in the Prefrontal Cortex by the trial before the G condition in analysis support hypothesis H2 and suggest more frontal activation for the switching from Novel to the Goal which is also concordant with Koechlin’s model of the frontal context attention control [5] and with Corbetta’s model lateralizing to the right hemisphere [20].

**Table 6:** Brain areas and statistical results of the 'distracted' subgroup (*n *= 6) with *p*< .03 and 1055 voxels activated for Novel and Goal stimulus vs Zero and Goal conditions— N.GvsZ.G

	Significant brain areas activated	Voxels with maximum *T*- value					Brodmann areas	Significant brain areas activated	Voxels with maximum *T*- value					Brodmann areas
	Positive difference	Statistics		Coordinates				Positive difference	Statistics		Coordinates			
		*T*	*p*	*x*	*y*	*z*			*T*	*p*	*x*	*y*	*z*	
*Frontal lobes*								*Frontal lobes*						
1	L PrecentralGyrus	4.11	.005	− 35	8	36	9	1 R InferiorFrontal Gyrus	3.75	.007	27	32	− 7	47
2	R MedialFrontal Gyrus	3.36	.01	18	37	24	9	2 R PrecentralGyrus	2.51	.027	48	− 11	10	13
	Parietal lobes							Parjetal lobes						
								3 L Precuneus	12.40	< .001	− 12	− 44	37	31
								4 R AngularGyrus	22.84	< .001	47	− 70	37	39
								5 R InferiorParietal Lobule	15.41	< .001	47	− 67	38	39,40
								6 R Precuneus	7.92	< .001	44	− 70	41	19,39
								7 R SupramarginalGyrus	5.96	< .001	62	− 46	30	40
*Temporal lobes*								*Temporal lobes*						
								8 L MiddleTemporal Gyrus	8.73	< .001	− 60	− 29	1	21,22
								9 L SuperiorTemporal Gyrus	6.08	< .001	− 54	− 27	1	21,22,41
								10 L TransverseTemporal Gyrus	4.16	.004	− 40	− 31	11	41
3	R CaudateCaudateTail	2.81	.019	33	− 39	3		11 R MiddleTemporal Gyrus	4.31	.004	52	− 63	29	39
4	**R SuperiorTemporal Gyrus**	3.32	.011	44	− 20	− 5	**22**	12 **R SuperiorTemporal Gyrus**	4.63	.003	38	− 32	16	13,**22**,41
*Occipital lobes*								13 R TransverseTemporal Gyrus *Occipital lobes*	3.34	.01	50	− 28	10	41
5	R FusiformGyrus	6.81	< .001	28	− 48	− 7	37	14 L Cuneus	20.87	< .001	− 15	− 78	29	18,19
6	R ParahippocampalGyrus	7.34	< .001	26	− 48	− 9	37	15 L Precuneus	18.92	< .001	− 7	− 57	27	31
								16 R Cuneus	6.21	< .001	14	− 89	30	19
*Limbic lobes*								*Limbic lobes*						
7	R AnteriorCingulate	3.26	.011	21	34	24	32	17 L CingulateGyrus	13.53	< .001	− 5	− 58	26	31
8	R ParahippocampalGyrus	5.41	.001	30	− 45	− 9	19,37	18 L PosteriorCingulate	14.32	< .001	− 6	− 58	24	31
	*Deep gray (Sub lobar areas)*							*Deep gray (Sub lobar areas)*						
9	R ThalamusPulvinar	6.41	< .001	9	− 30	9		19 L LentiformNucleus	3.24	.011	− 15	− 5	4	
								20 L LentiformNucleusLateralGlobus Pallidu	4.01	.005	− 15	1	5	
								21 L LentiformNucleusMedialGlobus Pallidt	3.18	.012	− 12	1	2	
								22 L LentiformNucleusPutamen	4.00	.005	− 18	1	3	
								23 L Thalamus	5.08	.002	− 9	− 9	1	
								24 L ThalamusMammillary) Body	3.23	.012	− 13	− 20	2	
								25 L ThalamusMedial DorsalNucleus	4.02	.005	− 7	− 20	3	
								26 L ThalamusVentral AnteriorNucleus	3.08	.014	− 16	− 8	12	
								27 L ThalamusVentral LateralNucleus	6.11	< .001	− 10	− 10	3	
								28 L ThalamusVentral PosteriorLateral Nucl	3.19	.012	− 17	− 22	1	
								29 L ThalamusVentral PosteriorMedial Nucli	3.42	.009	− 15	− 20	3	
								30 R Claustrum	8.56	< .001	30	− 15	16	
								31 R Insula	5.62	.001	43	− 17	5	13
								32 R LentiformNucleusLateralGlobus Pallidi	3.45	.009	18	− 1	3	
								33 R LentiformNucleusPutamen	5.97	< .001	29	− 12	13	
*Deep gray (Sub lobar areas)*								*Deep gray (Sub lobar areas)*						
								34 R Thalamus	18.2	< .001	9	− 24	1	
								35 R ThalamusMammillary Body	3.65	0.007	12	− 22	1	
								36 R ThalamusMedial DorsalNucleus	7.42	< .001	7	− 19	3	
*Additional regions*								37 R ThalamusVentral LateralNucleus Additional regions	3.29	0.011	14	− 14	3	
10	R Anterior LobeCulmen	4.30 .004	7 − 42 1					*Additional regions*						
								38 L MidbrainSubstania Nigra	5.21	0.002	− 14	− 21	− 6	
								39 R MidbrainRed Nucleus	3.36	0.01	6	− 17	− 6	
								40 R MidbrainSubstania Nigra	3.14	0.013	10	− 18	− 7	

Anatomical labels and associated *T* statistical values are listed. *t* scores from the omnibus analyses of 6 participants for each ROI are presented

Table 6 also shows the differences in the left and right Anterior Cingulate Cortex (ACC). This is consistent with the view of ACC in conflict monitoring [25], which is the previous context in our analysis.

## Discussion for contrasts, context to extend multimodal task

The first results discussed here focus on the 6 ‘distracted’ participant’s analysis which showed more significant brain activations than found for the whole group of 11 participants.

The analysis of these fMRI data (a) explored the effect of prior context across participants supporting H2 but only for ‘distracted’ participants; (b) explored novel response generators and simultaneous novel and target response generators relative to the standard goal condition supporting H1 but only for ‘distracted’ participants; (c) resulted in a larger recruiting neural response at the prefrontal cortex having less areas for standard goal stimulus and standard previous motor response and (d) attempted to find a possible explanation for the observed smaller than expected Novel sound ERP amplitudes. Last two analyses allowed having a grasp for modelling of auditory and motor function of the human brain (H3).

### RT results suggest that the novelty effect may vary between causing alerting and orienting

The RTs observed in the orienting subgroup were slower (20 to 70 ms) in the simultaneous novel and target (NG) condition suggesting that the focus of attention can be shifted with the introduction of a novel stimulus alongside the target in the mental representation of the auditory scene. In the literature we find this range of reaction times in orienting to alerting stimuli by Fan and colleagues [26]. According to Fan and colleagues, behavioural reaction time differences in alerting would be around 60 ms, orienting around 31 ms and conflict monitoring around 102 ms [26]. Brain areas of specific interest in the number parity decision task.

In the case of the parietal lobes: in the Z vs. G contrast the Right Precuneus were similarly activated only in this contrast; in the NG vs. G contrast the L/R Angular Gyrus, L/R Inferior Parietal Lobule and Left Superior Parietal Lobule (SPL) showed different activations only in this contrast for F-value difference; and in the N vs. G contrast the Left Precuneus showed similar activations only in this contrast while in the motor cortex the Right Paracentral Lobule showed different activations only in this contrast. Therefore, in the NG vs. G contrast, IPL and SPL showed different activations. Activation in the Precuneus (*p* ≤ 0.0005 uncorrected) is of interest because Precuneus is associated with reaching activity [27, 28]. Although in the present experiment the hand is not reaching different places, the selected finger (index or middle) is reaching the button for the task, the Goal and Novel stimulus showed an activation similar to the tendency to reach the novel, with different brain activations suppressing the button press in N vs. G more in the right Precuneus and allowing the button press in NG vs. G and Z vs. G in left and right Precuneus. Taking altogether the results for the contrast NG vs. G there is consistent with recent subdural electrodes in humans in the IPS, SPL and Precuneus for reaching a cup from a resting position [29].

On the temporal lobes: in the Z vs. G contrast the Left Sub Gyral area showed similar activations only in this contrast while in the different contrasts the L/R Transverse Temporal Gyrus (TTG) showed different activations. This is consistent with the result of the 750 Hz tone which activated more voxels in the medial area of the TTG, whereas the 2000-Hz tone activated more voxels in the lateral TTG [30]. Moreover, the Right Superior Temporal Gyrus (STG) has different activations in the different contrasts, which has been reported to be activated more by speech and frequency modulated tones [31]; in the NG vs. G contrast the L/R Angular Gyrus, Left Fusiform Gyrus, L/R Sub Gyral Hippocampus and Right Middle Temporal Gyrus showed different activations only in this contrast. Hippocampus and the different prefrontal areas activated during the task according to the presence of NG appeared by the presence of the novel when there is not an explicit sequence and having several conditions, in spite of Savalia and colleagues findings [9].

In the case of the occipital lobes: in the Z vs. G contrast the Right Fusiform Gyrus showed different activations only in this contrast; in the NG vs. G contrast the Right Cuneus/Precuneus Right Lingual Gyrus and Right Superior Occipital Gyrus showed different activations only in this contrast; and in the N vs. G contrast the Left Cuneus/Precuneus showed similar activations only in this contrast. FusiformGyrus activation reduces with repeated presentations, also when the performance of the participant is better [32]. In the present results, the L FusiformGyrus is more activated in the Novel than the Z and NG conditions, having clear differences at Goal as an object identification. However, there is no clear difference in the contrast of different conditions N vs G and N.G vs G.G. This supports the view that the orienting response is sensitive to the degree of familiarity with the experiment [33].

### Prefrontal cortex and motor responses in the preceding trial (H2)

Results showed that the Precentral Gyrus (PrG) motor area was activated differently in Z vs. G, N vs. G and NG vs. G contrasts. Activations were more ventral with relatively greater activations for the N condition (BA 43), and with relatively greater activations in different BAs in the NG vs. G contrast, in the left BA 6 for the NG condition and right BAs 4, 6 and 44 with relatively greater activations for the G condition. Moreover, taking into account the contextual contrasts, activations for Z.G vs. G.G contrast produced larger activation in the Right PrG (BAs 4 and 6) and for the N.G vs. G.G contrast had relatively greater activations for the N.G condition on the Left PrG (BA 4) and Right PrG (BA 4, 44 and 6). Therefore, overall all these results different prefrontal control is seen at PrG.

Although motor response is usually activated in the contralateral side, in this experiment the right hand was used in the parity decision task whilst some ipsilateral responses in the Left PrG were activated for N.G condition over G.G condition. Considering the change of the fundamental frequencies between N and G conditions, this left ipsilateral result to the right hand of response is consistent with frequency changes greater than 30 Hz observed for harmonic tones [34]. Thus, the Novel before a Goal makes more contribution to different motor area activations and similar activations than the NG conditions. Therefore, the ‘distracted’ participants showed a stronger attention to the task than to the motor control in N vs. G contrast and the motor control switch between N.G and G.G conditions, which is similar to the conflict motor control switch between NG and G conditions. Therefore, the motor response may be used in explaining the prefrontal control in the light of H2. This part of the discussion is expanded in the next part of the discussion which studies context from the point of view of the previous trial.

### Prefrontal cortex and context given by the immediately previous trial (H2)

Tables 3 and 4 show that there are more differences in NG.G vs. N.G than in G.G vs. N.G, consisting of more frontal areas and towards to the front as well for NG.G vs. N.G, which is consistent with the different frontal activations in the contextual approach of the hypothesis H2.

More insights derived from the results driven by hypothesis H2 are analysed in Table 7. This shows the comparison of the five contrasts analysed (first column). From Z.G vs. G.G to N.G vs. Z.G contrasts, it looks like the effect of a previous Novel stimulus is to increase the activation of the prefrontal areas. When both contrasts are compared to the N.G vs. G.G contrast, this increased activation of additional prefrontal areas is corroborated, and also the change of motor response results analysed in the previous section in the activation of additional prefrontal areas. In Table 7, when the first and third rows are compared with the fourth and fifth row, respectively, a similar increase of the number of areas in the prefrontal region is shown. Result suggested, in Table 7, when instead of G is NG part of the increased number of PFC areas is because of the recruiting of the brain areas closer to the ACC.

**Table 7:** Input/Output comparison of the number of Brain areas for the different contrasts explored

Contrasts	Previous input	Previous output	Current output						Table reported
	Previous stimulus	Previous Motor response	Number of frontal areas activated	Max frontal axis (mm)		Number of frontal areas activated	Max frontal axis (mm)		
				Left	Right		Left	Right	
Z.G vs G.G	Number vs number	not vs do	9	− 24	28	1	39	–	Table 2
N.G vs Z.G	Novel vs number	not vs not	2	8	37	2	–	32	Table 6
N.G vs G.G	Novel vs number	not vs do	9	41	37	–	–	–	Table 3
NG.G vs Z.G	Novel + number vs number	do vs not	6	52	61	12	22	41	Table 5
NG.G vs N.G	Novel + number vs novel	do vs not	6	-	18	10	50	56	Table 4

ACC activation was shown in both hemispheres (see Tables 7 and 8) related to NG.G (versus N.G and Z.G) and in the left hemisphere (see Table 3) related to N.G (versus G.G). First, this ACC activation is consistent with the view that the ACC facilitates control of attention [25]. These results showed consistency with conflict monitoring being more frontal and deeper for NG.G vs. N.G contrast, see Left ACC at (-10, 34, -10) mm and the Right ACC at (3, 30, 0) mm in Table 7). Alongside the comparison in Table 7, these results in frontal areas are not only consistent with the prefrontal control proposed by Koechlin and colleagues [4], but the R SMG is also consistent with the model of control of attention proposed by Corbetta and colleagues [7].

### fMRI for ‘distracted’ participants showed left and right brain areas for contextual conditions in the attention model (H1 and H2)

First, the results of the Z.G vs. G.G contrast showed different right parietal activation and no different occipital areas as the signature of this contrast. The results are summarized in the graphic in Fig. 4 and they have shown consistency with the visual stimulus-driven attention network model of Corbetta and Shulman [7] as shown for the left hemisphere in the dotted rectangle in yellow. Although the positive contrast results are not exactly consistent with the reorienting of attention of Corbetta and colleagues [20], the activations in Brodmann Areas 7, 19 and 39 may be related to activity in the R IPs. However, the FEF is not clearly activated. In addition, the negative contrast only showed significant activation of the left Medial Frontal Gyrus without a clear different activation of the control of attention for the G.G condition. Of course, this can be explained because the current trial (G) has mostly the same properties of the frequently previous trial type (G). These interpretations suggest that the Z.G is evoking an interaction of the stimulus and goal-driven network differently to the pattern orienting of attention, while the IPs is suggested to be related to BAs 7, 19 and 39 (see dotted rectangle in green).

**Fig. 5:**
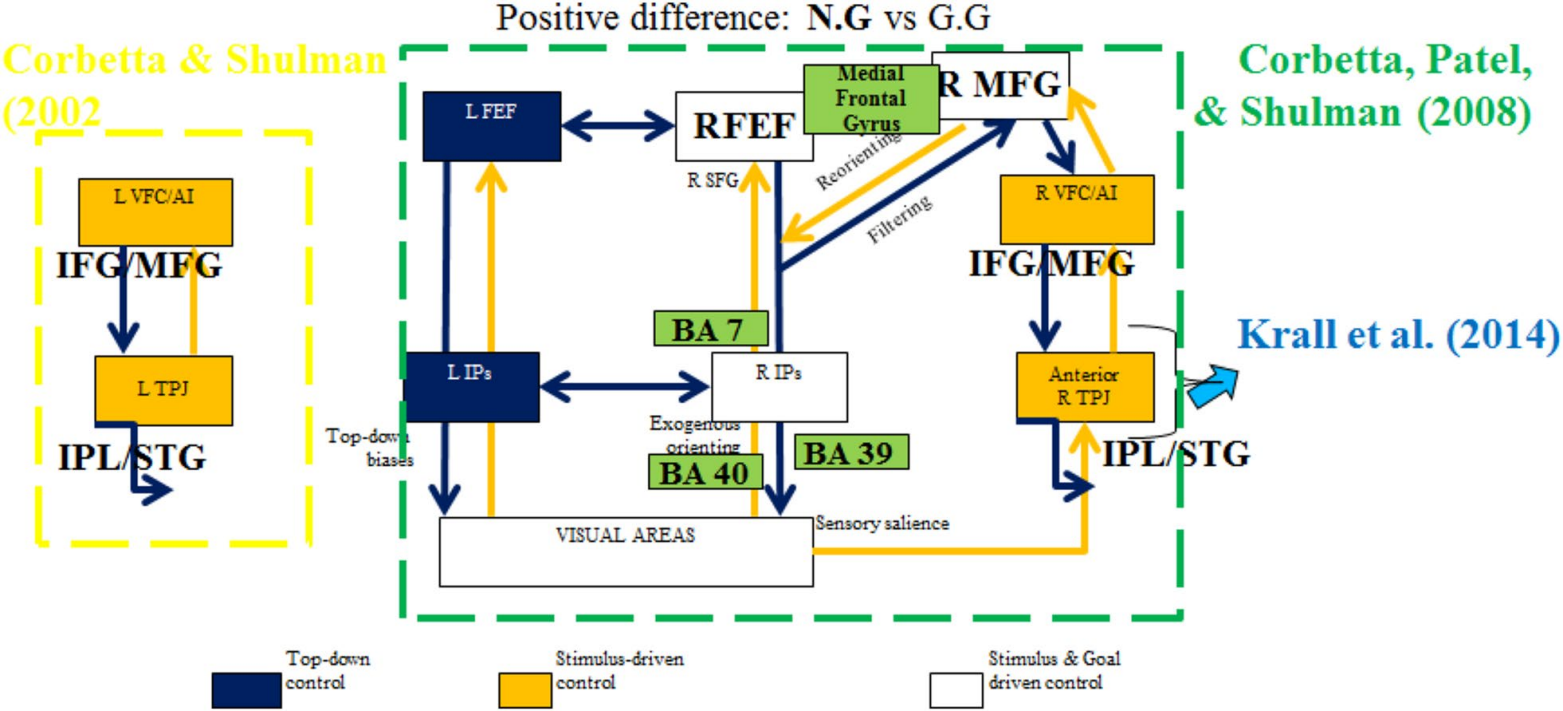
Positive differences of the brain regions for the contrast N.G vs. G.G, showing an interaction between Filtering and Reorienting mode of attention. Several attention areas on the Right hemisphere were with relatively greater activations to the NG condition

Second, when the N.G and G.G contextual conditions are more involved in a different frontal control of attention: the results of the N.G vs. G.G contrast showed different left and right parietal activation and no differences in occipital areas as the signature of this contrast. The results are summarized in the graphic in Fig. 5. The results support right and left (see dotted rectangle in yellow) hemispheres in the stimulus-driven attention network of Corbetta and Shulman [7] suggesting the control of attention in the N.G sequence. Although, the positive contrast results are not exactly consistent with the reorienting of attention of Corbetta and colleagues [20], but the Brodmann Areas 7, 40 and 39 may be enclosing the activity in the R IPs. Further, the negative contrast only did not show significant activation of the cortex; again, this can be explained because the current trial (G) has mostly the same properties of the previous trial (G). These interpretations suggest that the N.G is evoking an interaction of the stimulus and goal-driven network similar to the pattern orienting of attention (see dotted rectangle in green).

Finally, right lateralized Thalamus in the Pulvinar has shown significant response at novel response (Tables 5, 7, 8) also bearing the prior stimulus (Tables 11, 12) as well as no motor response (Table 6) or previous motor response (Table 7). This result is concordant with the finding of different management on Pulvinar on selective attention [35], here under different conditions modulation was found according to different prefrontal areas. Moreover, this modulation would be concordant with the finding that the ventrolateral Pulvinar receives inputs from the prefrontal cortex, helping in modulation of visual processing and attention [36]. Although our methods in the current study are not sensitive enough to examine ventrolateral Pulvinar, we have found that it can encode prior context either auditory signals or motor responses that can be explore to study lateral geniculate nucleus (LGN) to top-cortical areas and from these areas to Pulvinar-like structures [37].

### fMRI and ERP comparison and the anterior cingulate cortex

Comparing fMRI and ERP results in the ‘distracted’ subgroup: (a) the Anterior Cingulate Cortex (ACC) is not activated differently between Z and G conditions (Table 2) and the ERP deflection around 200 ms, biased for Z condition negatively to the left frontal electrode F7 and positively to the right frontal electrode F8 in Additional file 1: Figure S1; (b) Right ACC is activated differently between NG and G conditions (Additional file 1: Table S4) being more frontal for NG condition in the right ACC (BA 32) and more posterior for the G condition (BA 32) and the negative ERP deflection around 200 ms in the right electrode F8 (in Additional file 1: Figure S1) and stronger Left ACC is activated differently between NG and G conditions (Additional file 1: Table S4) being with relatively greater for the NG condition in the left ACC (BA 32) and the negative ERP deflection around 200 ms is stronger to the left frontal electrode F7 (in Additional file 1: Figure S1); (c) difference between N and G conditions (Additional file 1: Table S1) and no clear difference around the ERP at 200 ms (F7 and F8 in Additional file 1: Figure S1). These results suggest that ACC is linked to N200 for NG condition in both hemispheres. On the other hand, in the N vs. G contrast positive and negative activation differences in ACC were observed and no clear ERP different deflections around 200 ms, namely MisMatch Negativity. This analysis is consistent with the view of N200 and ACC in conflict monitoring studies [25]. However, but, because of MMN, it is not clear about the Novel effect.

Moreover, ACC activation was shown to be different across the other contextual contrasts (Z.G vs. G.G, N.G vs. G.G, N.G vs. NG.G, NG.G vs. Z.G and N.G vs. Z.G) and the relatively greater activation was shown not only for novel, but also for Zero condition. Therefore, ACC relative activations were sensible to contextual changes depending on Goal (G), Non-Goal (Z and N) and Novel (N and NG) signals.

In the ‘distracted’ participants, the contrast between NG.G and N.G was evaluated for the ACC. Results showed relatively greater activation for the N.G condition in the Left (BA 32) and Right (BA 24) ACC. This suggests that ACC produces different activations depending on the previous context for stimulus-driven network and the conflict monitoring effect. When the contrast between NG.G and N.G conditions in ‘all the participants’ was evaluated, there were no significant differences in ACC activation and this suggests that ACC in the alerting state does not produce different activations for the different Novel trials presented before the current Goal trial. These differences between the ‘distracted’ and the ‘all participants’ would explain the difference of the analysis of the ERP at N200 in Potter’s study [38] and ACC in fMRI in the present analysis of the ‘distracted’ subgroup.

Another possible comparison would be a further eye field activation in fMRI and beta waves in EEG such as was found for higher arousal levels [39]. The present analysis may accommodate the role of the FEF in attention when the Corbetta’s model of attention is considered. Therefore, a further limitation in the present analysis is that this was the third task in the participants and possibly the results for FEF in the ‘distracted’ participants added to the inhibition of return for Z vs G contrast were related with the arousal level to keep the answer to the task in the auditory attention task.

In practical use to add in this discussion, this experimental discussion may have a theoretical extension to be used by BCI systems that involve the management of neural network and learning systems architectures. This was addressed in the following conclusion.

Limitations of 12 participants were compensated by a FDR analysis (similar number in fMRI statistic comparison by Nichols [40]) and bearing in mind current theory of attention and a similar auditory paradigm, which explored context with EEG in schizophrenic participants [3].

## Conclusion: improvement of modelling novel response due to previous motor response

**Fig. 6:**
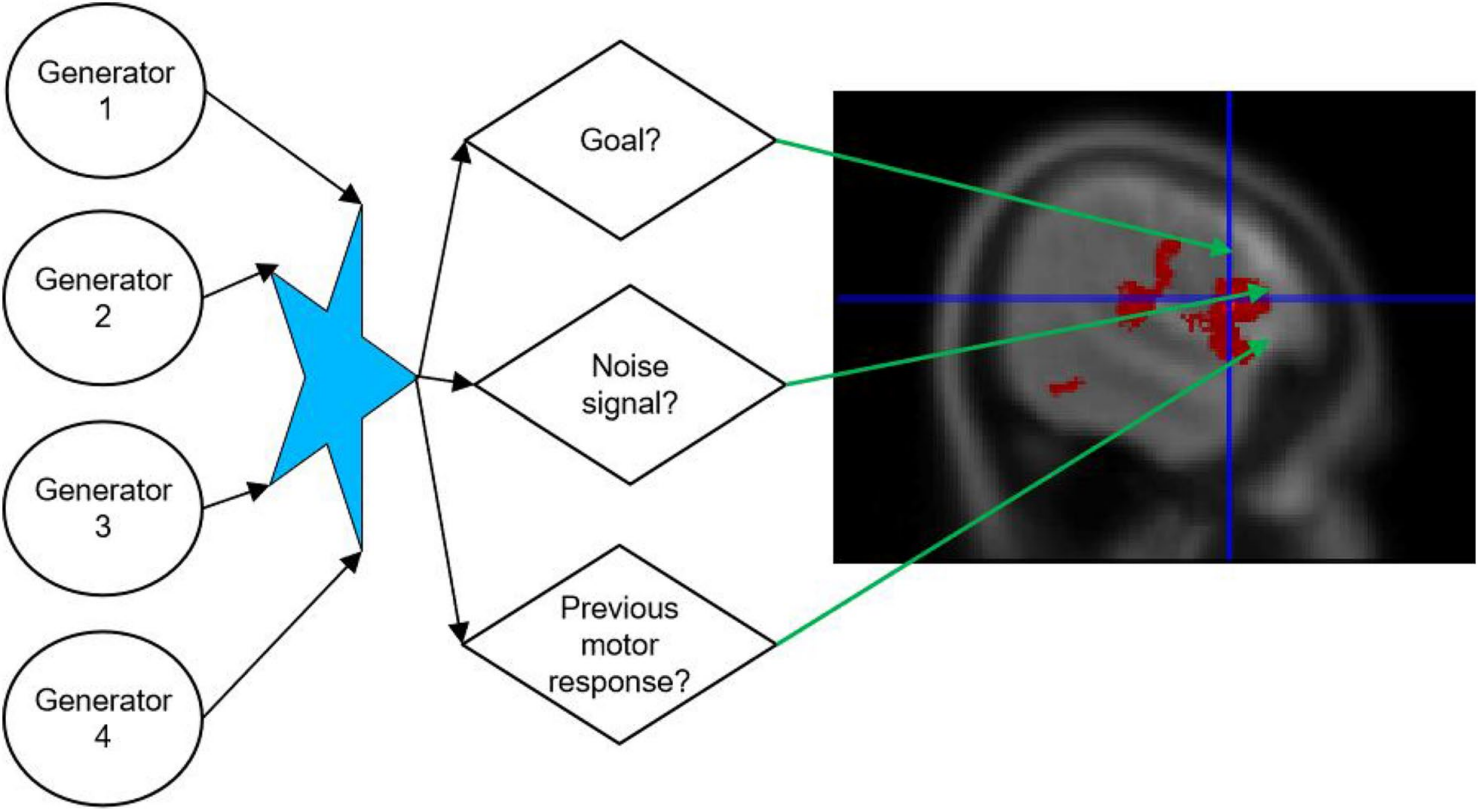
Modelling of number of prefrontal areas activated by several generators, where motor response modulated brain areas activated

Given results and discussion, the sequence of stimulus studied has shown different activation of the hippocampus areas which have been in favour of the theory or cortical and subcortical loop for sequence proposed by Savalia and colleagues [9]. Moreover, the present results have reported when a sequence is interrupted by a novel (simultaneously) the subcortical loop with the hippocampus is also activated. This has extended Mugruza-Vassallo and Potter studies of temporal stimulus sequence with EEG [3] to fMRI brain regions and following their analysis and extension of management of novel stimulus modulated by the previous motor answer a model is proposed in Fig. 6 solving part of the puzzle proposed by Livnet and Zador [14]. These consistencies make it of interest to explore another experiment to study the EEG results in more detail and combine with the fMRI analysis to seek for the explanation of these partial consistencies.

Bearing in mind eye movement research in response to an auditory experiment has shown results in pupil dilation response [41], the present findings on motor modulation of attentional processing would be extended by a broader motor response. Moreover, the model would modify the Information Dynamics of Thinking (IDyOT) model for language and music of Forth and colleagues [42] may bear in mind previous motor response and unexpected external stimulus. Forth and colleagues proposed a mechanism for predicting when a perceptual event will happen, given an existing sequence of past events, which may be musical or linguistic [42].

Evolutionary multitasking computation [19] maybe best based on multi-objective optimization of cortical prefrontal cortex for different incoming stimulus employing stimulus features for objective functions (f) for vectors of decision variables (y) in the search space (Y) following Eq. 3, considering 4 conditions:

3 $${\text{maximize}}(y \in Y) \, f\left( y \right) \, = | f_{1} \left( y \right) \, ; \, f_{2} \left( y \right) \, ; \, f_{3} \left( y \right); \, f_{4} \left( y \right)|.$$

Then for *K* = 4 different tasks (T1, T2, T3, T4) the MOP in terms of the populations would follow Eq. 3, but bearing in mind the different responses due to previous motor command. In this way *f*_*k*_(*y*) will depend on the neural processing of previous motor response *y*(*m*_(*nT−T*)_) and the current motor response *y*(*m*_(*nT*)_), as seen in (4):

4 $$f_{k} \left( y \right) \, = \, g_{k,1} \left( {y\left( s \right)} \right) \, g_{k,2} \left( {y\left( {m_{(nT)} ,y\left( {m_{(nT - T)} } \right)} \right)} \right).$$

Also bearing in mind our “inhibition of return” results, they influence on the number of prefrontal areas modulated. Therefore, an additional input would be needed to maximize decision variables going for at least *m* = {0, 1}, 0 for no motor response and 1 for motor response in (5). This would be valid for 2-oddball tasks (e.g. [24]):

5 $$\begin{array}{*{20}c} {} \\ {{\text{max}}(y \in Y) } & { \sum_{k = 1}^4 \int z f_{k} \left( z \right) \cdot } & {\left[ {\sum_{j = 1}^4 w_{jk} ,_{m(k - 1)} \cdot p_{j,m(k - 1)} \left( z \right)} \right] \cdot {\text{d}}z} \\ {\left\{ {w_{jk} .p_{j} \left( z \right)} \right\}} \\ \end{array} .$$

Therefore, we may have *f*_*1*_(*y*) relying on G condition, as well as *f*1(*y*), *f*2(*y*), *f*3(*y*), *f*4(*y*) relying on G.G, Z.G, N.G and NG.G from Eqs. 6 and 7. The power of analysis (7.a, 7.b, 7.c, 7.d) is better than for only one condition (6.a). In (6) prior context would not be reached by almost any *gg*_*i,j*_ in particular:

6.a $$ff1\left( y \right) \, = \, gg_{1,1} (y(s = G)) \, gg_{1} ,_{2} \left( {y\left( {m\left( {nT} \right),y\left( {m\left( {nT - T} \right)} \right)} \right)} \right),$$

6.b $$ff_{2} \left( y \right) \, = \, gg_{1,2} \left( {y\left( {s = Z} \right)} \right) \, gg_{k,2} \left( {y\left( {m_{(nT)} ,y\left( {m_{(nT - T)} } \right)} \right)} \right),$$

6.c $$ff_{3} \left( y \right) \, = \, gg_{1,3} \left( {y\left( {s = N} \right)} \right) \, gg_{k,2} \left( {y\left( {m_{(nT)} ,y\left( {m_{(nT - T)} } \right)} \right)} \right),$$

6.d $$ff_{4} \left( y \right) \, = \, gg_{1,4} \left( {y\left( {s = NG} \right)} \right) \, gg_{k,2} \left( {y\left( {m_{(nT)} ,y\left( {m_{(nT - T)} } \right)} \right)} \right).$$

An example of the power of analysis by *ff*, we may have Z.G vs G.G and on other hand N.G vs NG.G where the Pulvinar was activated as well employing different parts of Eq. (7), where prior context may be considered by *gg*_*i,j*_ in particular can account prior context contrasts, being different from inhibition of return, standard stimulus and both different way of novel stimulus:

7.a $$f1\left( y \right) \, = \, g_{1,1} \left( {y\left( {s = G.G} \right)} \right) \, g_{1,2} \left( {y\left( {m\left( {nT} \right),y\left( {m\left( {nT - T} \right)} \right)} \right)} \right),$$

7.b $$f2\left( y \right) \, = \, g_{2,1} \left( {y\left( {s = Z.G} \right)} \right) \, g_{2,2} \left( {y\left( {m\left( {nT} \right),y\left( {m\left( {nT - T} \right)} \right)} \right)} \right),$$

7.c $$f3\left( y \right) \, = \, g_{3,1} \left( {y\left( {s = N.G} \right)} \right) \, g_{3,2} \left( {y\left( {m\left( {nT} \right),y\left( {m\left( {nT - T} \right)} \right)} \right)} \right),$$

7.d $$f4\left( y \right) \, = \, g_{4,1} \left( {y\left( {s = NG.G} \right)} \right) \, g_{4,2} \left( {y\left( {m\left( {nT} \right),y\left( {m\left( {nT - T} \right)} \right)} \right)} \right),$$

7.e $$f_{5} \left( y \right) \, = \, g_{5,1} \left( {y\left( {s = Z} \right)} \right) \, g_{5,2} \left( {y\left( {m_{(nT)} ,y\left( {m_{(nT - T)} } \right)} \right)} \right),$$

7.f $$f_{6} \left( y \right) \, = \, g_{6,1} \left( {y\left( {s = N} \right)} \right) \, g_{6,2} \left( {y\left( {m_{(nT)} ,y\left( {m_{(nT - T)} } \right)} \right)} \right),$$

7.g $$f_{7} \left( y \right) \, = \, g_{7,1} \left( {y\left( {s = NG} \right)} \right) \, g_{7,2} \left( {y\left( {m_{(nT)} ,y\left( {m_{(nT - T)} } \right)} \right)} \right).$$

Here (7) would be best according to the results of the condition of the trial immediately prior to the current trial as this fMRI analysis has shown significant results: Z.G vs. G.G, N.G vs. G.G, NG.G vs. Z.G, NG.G vs. G.G, and N.G vs. Z.G. From (*7.a-d*) contrast difference found may be accounted by contrast (*f*_*2*_(*y*)*, f*_*1*_(*y*))*,* contrast (*f*_*3*_(*y*)*, f*_*1*_*(*y*)*)*,* contrast (*f*_*4*_(*y*)*, f*_*1*_(*y*))*, *contrast (*f*_*3*_(*y*)*, f*_*2*_(*y*))*,* contrast (*f*_*2*_*(y), f*_*3*_(*y*))*.* An extension of this proposal clearly considers features on signals, where features can be stimulus properties as well.

Also, EEG research may use formulation by (7) on findings considering previous and later interventions on videogames on spectral ERP for fortress hits, rare tones (inside and outside the game), and mine appearances [43]. Limitation here is for a variety of complex and non/complex tasks maybe worked [44].

Main limitation for this proposal is to ignore possible conflict when one tends to think about a bad previous response. In the present experiment, errors were less than 10% in most of the participants, moreover not different for having more contextual variable are not accounted by *f*_*5*_(*y*) (equivalent to *ff*_*2*_(*y*)), *f*_*6*_(*y*) (equivalent to *ff*_*3*_(*y*)) and *f*_*7*_(*y*) (equivalent to *ff*_*4*_(*y*)), of course more experiments should be done to account properly how multitask and prior context affects other conditions. This would open to study motor response with error response in decision-making responses and improve current learning systems in BCI.

This motor response recruiting prefrontal areas would support the idea that the learning modelling of the task has not a linear function influenced by the learning parameter, the greater the maze size for goal-task the more steps to get an optimal pathway [45]. Moreover, the model proposed may help in the future to find compensatory effects in Parkinson’s disease by recruitment of more brain area in the prefrontal cortex and extend not only the present work but also work of Martin and colleagues at planning and executing motor employing different hands might be studied simplifying their experiment with an additional condition of motor planning [46]. In this way dopamine pathway can be revisited, having (7) in frequency may help to study beta frequencies in Parkinson at synchronization of the basal ganglia (BG) and thalamus wit cortex [47] as well as a less studied dopamine interpretation for anaemia in children [48]. Impaired motor function would be described as a change con *g*_*i,2*_ (*i* = *{1, 2,.., 7*) and current treatment experiments such as DOPA-ON and ON-Deep Brain Stimulation where the higher duration the longer beta peaks in patients OFF medication (peak width at half height, 106 ms) compared to controls (peak width, 46 ms) [49].

Limitation for motor response in the present research was about the extension of motor control in the research area of “coordination”. Marsh and colleagues pointed perception–action systems come to task of ecosystems [50], therefore considering multi-stability for social behaviour and multiple participants present in several real setting multitasks [51]. Participants are believed to not only use dopamine pathway to social rewards, but also to context dependence in complex environment where new selections are done base on dynamic interaction of task [52]. Although the present work has given a better insight of auditory multitask and motor control, it did not reach a real setting multitask, therefore more work should be done to use multitask in perception–action ecosystems in real world.

Another area of further test may be on multitask switching on dyslexia, considering our results mainly on right Pulvinar which is close to LGN, our experimental results suggest an asymmetry for brain processing. Bearing this result on our auditory number parity decision task, language multitask switch may be explored as well, as LGN asymmetry was reported by proton density with MRI recently by Giraldo-Chilca and Schneider [53]. Moreover, in this study, the different modulation of brain areas in the PFC and its concurrent Pulvinar activation may be related to different “coordination through the Pulvinar’s involvement in up-regulating activity” [54]. Therefore, current research would be extended by an experimental design using EEG and fMRI to study PFC and Pulvinar interaction with LGN different frequency bands as a Deep Predictive Learning [37, 55, 56] as well as TMS has been suggested to improve this understanding in dyslexia as well [37]. On the other hand, a possible extension of the present work may be extending cortical–pulvinar interaction described by Kanai and colleagues [57] in terms of some of the equations developed here, namely (7). Possibly extension of the present experiment for modelling may be used to extend findings on two choice tasks.

Finally, bearing in mind discussion of multitask experiment [3] discussed in use of person identification with reliable decoders [2] and re-identification using different visual views [1] in systems with different interfaces. These interfaces may involve not only EEG, but also precise electrode positions inferred or combined with fMRI or fNIRS as occipital images, as the present work suggests. Moreover, prior context in auditory signals has been related to probability is related in auditory judgment with Hidden Markov Models [58] and therefore to attention and decision-making, next step to setup probabilities in the present research is to study parallel judgment as visual 2D, 3D and Augmented reality is been doing recently with Markov chains [59, 60].

## Supplementary Information

**Additional file 1.** EEG results and typical fMRI contrasts.

## Data Availability

Program, source code and contrast files in SPM are accessible at cmugruza@yahoo.com (we are heading to share in some workspace on https://github.com/cmugruza/fMRIpriorContext). The human head MRI data set was donated under the condition of anonymity during the filling consent inform of each participant.
