# Supplementary material for: Prior context influences motor brain areas in an auditory oddball task and prefrontal cortex multitasking modelling
Source: Brain Inform. 2021 Mar 21;8(1):5. doi: 10.1186/s40708-021-00124-6 (PMC7982371; doi:10.1186/s40708-021-00124-6)
Supplement: Supplementary file 1 — Additional file 1. EEG results and typical fMRI contrasts. [file 40708_2021_124_MOESM1_ESM.docx]

**Additional file: EEG results and classical fMRI contrasts**

Carlos A. Mugruza-Vassallo ^1^*, Douglas D. Potter ^2^, Stamatina Tsiora ^3^, Jennifer Macfarlane ^4^, Adele Maxwell 2

^1^ Grupo de Investigación de Computación y Neurociencia Cognitiva, Facultad de Ingeniería y Gestión, Universidad Nacional Tecnológica de Lima Sur, Lima, Peru

^2^ Neuroscience and Development Group, Arts and Science, University of Dundee, Dundee, United Kingdom

^3^ School of Psychology, University of Lincoln, Lincoln, United Kingdom

^4^ NHS Tayside, Ninewells Hospital, University of Dundee, Dundee, United Kingdom

Correspondence should be addressed to Carlos A. Mugruza-Vassallo; [cmugruza@yahoo.com](mailto:cmugruza@yahoo.com)

# Introduction

Among the multimodal techniques utilised to study the brain, EEG offers high temporal resolution but limited spatial resolution while fMRI offers high spatial resolution but lower temporal resolution. Logothetis and colleagues (2001) carried out seminal work on monkeys with the study of simultaneous intracranial recordings and the blood-oxygen-level-dependent (BOLD) fMRI responses on the visual cortex at 4.7 T. With intracranial recordings the local field potentials (LFP) were obtained. LFP reflects mostly a weighted average of synchronized dendro-somatic components of the input signals of a neural population. Therefore the LFP has a similar physiological basis to scalp EEG. Significant correlations (*r*) between the LFP and the BOLD responses were found; on the one hand *r* > .5 was found at frequencies greater than 15 Hz and, on the other hand, *r* < .3 was found for frequencies smaller than 8 Hz (Logothetis, Pauls, Augath, Trinath, & Oeltermann, 2001). EEG and fMRI can therefore be usefully combined in separate EEG and fMRI recordings with the same experimental design, but also in simultaneous EEG and fMRI recordings (e.g., Debener et al., 2006; De Martino, de Borst, Valente, Goebel & Formisano, 2011). In an auditory oddball task, Horovitz and colleagues (2002) manipulated the probability of the goal tone using a variable number of non-goal standard tones ranging from 12 to 51 (i.e. local probabilities from 7.69 % to 1.92 %) between consecutive goal tones in a long experiment. The task mainly consisted of non-goal standard tones of 1000 Hz and goal tones of 1500 Hz. They found an increase in P300 amplitude in Pz while BOLD-signal changes increased in the same direction in the SupraMarginal Gyri, Thalamus, insula and right Medial Frontal Gyrus (Horovitz, Skudlarski, & Gore, 2002).

From the ERP deflections: N1 is an earlier deflection and has strong generators in the auditory cortex (Naatanen & Picton, 1987; Picton et al.*.*, 1999). Mulert and colleagues (2004) study the activation of temporal, parietal and frontal regions in an auditory oddball task, by recording EEG responses outside as well as inside the scanner (simultaneous EEG/fMRI). The task mainly consisted of non-goal standard tones of 800 Hz and goal tones of 1300 Hz. They reported that although the P300 component was not significantly different when recorded outside or inside the scanner, the N1 component was found to be significantly smaller and of longer latency inside the 1.5 T scanner (Mulert et al. 2004). Consistent with previous results reviewed by Corbetta and Shulman (2002), studies suggest that the target detection (reflected in the P3b component) network comprises the frontal areas, the insula and the temporoparietal junction (TPJ) [7].

The original aim of the experiment described in this study was to pilot an auditory attention paradigm with simultaneous EEG and fMRI at 3 Tesla during the technical development phase of the newly installed scanner. The data were collected using a simplified version of the number parity decision paradigm (see Table 1 in main article). This study was carried out prior to the analysis described by Mugruza-Vassallo & Potter in 2019 [3] and the design of this experiment was therefore not informed by the outcome of contextual analysis between a pair of sounds is greater for schizophrenic patients. This experiment was carried out as part of a series of experiments where participants in the first block were required to lie still in the scanner for 5 minutes (resting state), in a second block they completed a visual working memory (N-Back) task, in a third block they completed 400 trials of the auditory number parity decision task and in the last block they completed a conditioning paradigm. The basic design of the auditory number parity decision task consisted of either a number (goal driven) where participants had to make a forced choice “odd” or “even” response, or a number with a simultaneous laterally presented novel stimulus (goal + stimulus driven) as ‘go’ trials and either the number zero or novel-only (stimulus driven only) as ‘no-go’ trials. The original aim of this study was to dissociate activation of the stimulus-driven and goal-driven networks (SDN and GDN) by comparing the hypothesised goal-driven parity decision trials to the hypothesised stimulus-driven distractor trials in the analysis of simultaneous EEG and fMRI data. Preliminary grand averaged ERP waveforms from 5 of 12 participants are illustrated in Figure S1.

The preliminary results indicate the presence of several distinct ERP deflections at different electrodes. Right lateralized electrodes (*e.g.,* T8) dissociate ‘go’ from ‘no-go’ trials whereas a large positive deflection at ~600 ms at Pz in all the number decision conditions showing an auditory P3 complex. Electrodes PO7 and PO8 in the no-go ‘Zero’ condition allow one to differentiate the ‘slow wave’ associated with behavioural response (P650) from the P3b associated with the resolution of stimulus processing and updating of working memory (P550). In the case of ‘Novel Only’ stimuli, only a small right lateralized P3a response at ~550 ms was observed (Potter et al., 2010). This last result is consistent with Corbetta and colleagues’ argument with regards to the activation of the stimulus-driven part of the attention reorienting network without activation of the goal-driven system and attention orienting [19]. Moreover, exploring the ERP among 5 participants leads to a greater P1 component for simultaneous novel and goal and smaller ERP deflections for novel sounds (Potter et al., 2010).

**EEG Data Analysis**

Pre-processing was conducted first using Analyzer software (Brain Vision, LLC). MR artifacts were corrected using an artifact template based on 30 volume acquisitions with a further low pass filter at 70 Hz and sampling frequency was downsampled to 256 Hz. The cardioballistic artifact was corrected using a template based approach. Data were then exported to EEGLAB and eye blinks and other artifacts were rejected using Independent Component Analysis (ICA).

In the introduction, preliminary analysis demonstrated the presence of identifiable ERPs (Potter et al., 2010). For this experiment, the G condition has 250 trials and the other 3 conditions have 50 each one, this reduces the noise near to root square of 250 (≈ 15.8) in the G condition and similarly around ≈ 7.1 in the other 3 conditions. We were then confronted with the problem of finding reliable single trial ERPs, because of having too few participants (n = 5) in Potter’s analysis or all participants (n = 12) in the recordings of the present study. These numbers result in a reduction of the noise in the few participants around ≈ 2.2 or a reduction of ≈ 3.5 in the all participants. This noise reduction is added to the high electrical noise on the EEG recordings induced by the high magnetic field of the scanner. Furthermore, these noises affected the processing of the cardioballistic rejection. To be able to know whether we can resolve this problem, the EEG recordings were filtered with a low-pass at 70 Hz. Subsequently, raw EEG data and filtered EEG data were compared to find out whether the quality of noise on the EEG recordings was sufficiently reduced. Following the above procedures, EEG data were analyzed using EEGLAB (Delorme & Makeig, 2004) and Matlab in-house scripts. Eye-movements and artifacts were removed using an independent components analysis (ICA). Data was then filtered using a high-pass filter at 0.75 Hz and epoched from 300 ms before stimulus onset to 900 ms after stimulus onset. A baseline correction was then applied. Epochs were then checked for trials with excessive peak-to-peak deflections, amplifier clipping, or other artifacts. The EEG signal was then analysed in a single participant to determine whether the data were of sufficient quality to produce single trial averages across-participants. Because of the poor quality of the EEG data, the calculation of sound properties and P300 peaks followed by bootstrapped correlations and False Discovery Rate was not used.

## EEG Results

EEG comparison between the raw EEG signals and the filtered signals at 70 Hz were carried out for all the participants. To enable comparison, the EEG difference was plotted with threshold amplitude of 300 uV (10 times the expected maximum P300, *i.e.* 10 x 30 uV). Noise amplitude was calculated as the maximum amplitude of the 400 ms period after the stimulus (S2) was presented.

*Figure S2* shows in the participant TD04 a specific example of the variability in time of the noise generated by the recording system. For example, channels F1 and F2 are affected and noise is changing with time, while other channels such as Fz and Cz are not affected in the scale of 300 uV. Moreover, more than 200 trials did show problems of active auditory signals over electrodes. On the other hand, *Figure S3* in the participant TD05 shows a specific example of the variability in time of the noise generated only in some electrodes by the recording system. For example, channels T7, T8, CP5, TP10 and C1 are affected and noise is changing with time, while other channels such as Cz and Pz are not significantly affected in the scale of 300 uV. In addition, the signals on channels Fz and AF4 are greater than 300 uV in amplitude in all trials. Moreover, 65 trials (of 400 trials) did showed problems of active auditory signals over electrodes.

Our results showed that the noise was either (1) strong and localized in some electrodes changing slowly over time or (2) almost all electrodes were initially noise-free up to some point in time when variations in noise were increasing for many of the electrodes.

After the analysis of the different EEG datasets and cardiobalistic rejection the data were epoched for one of the clearest datasets of the 12 participants at electrodes Cz and Pz (TD05).

In this single subject EEG data (TD05), a further cardioballistic rejection was run and grand average ERPs were computed. The grand average ERPs relative differences are shown in *Figure S4*. It can be seen that after artifact removal there is limited evidence of an ERP. Although it was expected that ERPs in channels Cz and Pz would be evident (along other channels, i.e. F7 and T7), there are no clear differences between condition in Cz and Pz and possibly novel noise introduce a different spectral content (see first 300 ms in Fz channel). Therefore, this data processing was not good enough to proceed further with the single trial EEG analysis across all participants for the purpose of predicting fMRI signal fluctuations.

After cardioballistic correction and artifact rejection using ICA the EEG was averaged. ERP plots indicated that there was insufficient good signal for single trial analysis and it was decided not to proceed further with the single trial EEG analysis across all participants or attempt to use this information as a predictor in the fMRI analysis.

Because of the difficulties of the noise levels in the EEG datasets it seemed impractical to attempt single trial averaging across participants and it was decided to analyse the fMRI data independently. No further analysis of the EEG was therefore attempted.

## EEG Results suggest that the noise is coming from sound stimulus presentation

According to Debener’s experimental studies at 1.5 T, 3 T and 7 T, the higher the magnetic field of the scanner the higher the electrical noise on the EEG recordings (Mullinger, Debener, Coxon, & Bowtell, 2008). The problem of obtaining several trials for ERP measures using the MRI machine has been reported indirectly. For example, Mulert and colleagues in the simultaneous EEG and fMRI experiment employed 315 standard trials and 75 goal trials to get reliable ERPs and through the use of air tubes to deliver sounds (Mulert et al., 2005). In the present study, obtaining single trial ERPs proved to be an unrealistic goal for a number of reasons. A significant problem was that the sound stimuli that were being played through electrostatic headphones induced an artifact of similar size to the EEG signals in the first few hundred milliseconds of each trial.

The number of trials for single trial average ERP is 6 for the ‘distracted’ group and 10 for all the participants, i.e. reduction of noise around 2.45 times in the ‘distracted’ group and 3.16 times for the all participants. On the other hand, the number of trials for the ERPs in Potter’s (2008) preliminary analysis was 5 participants at 50 trials per each, giving a total of 250 trials, i.e. a reduction of noise around 15.8 times.

The quality of the recordings was variable in this first ever simultaneous EEG/fMRI recording in this lab and reliable removal of scanner and cardio ballistic artifacts proved to be highly challenging.

Yan and colleagues simulated EEG artifacts aiming to explore Debener’s results in artifacts in EEG signals at 3 T of MRI environment. First, a blood inducing Hall voltage was analysed, and EEG artifacts around 200 uV were observed in the left and right electrodes (F7 and F8) of different amplitudes in the first few trials. Moreover, when slow head movements were simulated, EEG artifacts of several hundred of uV in amplitude were observed (Yan, Mullinger, Geirsdottir, & Bowtell, 2010). Although, we did not considered the possible source of the pulse artefact, our results pointed to artefact of several hundred of uV after using an average template. Therefore, based on the EEG results (section 3.3.2), we suggest re-formulating the filtering of the EEG data bearing in mind the difference between filtered and non-filtered signals and the source of the pulse artifact studying head movements in the simultaneous EEG and fMRI experiment.

Originally our approach sought to link the analysis of individual items to the general linear model in SPM, using linear modelling under EEGLAB (LIMO in Pernet et al., 2011), in a similar analysis to that carried out by Debener and colleagues (Debener et al., 2006) in which they used elements of the ERP response as predictors of fMRI signals. In the present experiment, the number of trials is 400 and the noise in EEG signal responses without auditory effects over electrodes in some cases is less than 100 trials. Added to the change of the variable artifacts at each trial concordant with the problems reported in other studies (e.g. Yan et al., 2010, Debener et al, 2007) the present number of trials of around 100 is small in order to get enough good trials to use the second technique LIMO (explored in Mugruza-Vassallo & Potter, submitted) as carried out in the only EEG experiment with both control and schizophrenic patients with 600 trials and more than 500 effective trials for each participant. Further, in order to use the single trial ERP and to analyse the contextual control of attention, more participants would be needed to reduce the noise in the EEG average across-subjects. Therefore it is proposed to expand the experiment to up to 25 participants to reduce the noise 5 times, which is slightly greater than the number of participants in the experiment with controls and schizophrenics as discussed in Mugruza-Vassallo & Potter (submitted).

## fMRI Results suggest STG role in responding to target stimuli

Based on previous findings in the Amygdala and anterior Superior Temporal Gyrus (STG) of the removal or damage in these regions (Johnson, 1988, 1989; Polich & Squire, 1993), Kiehl and colleagues suggested the “adaptive reflexive processing” of some brain areas when they are required. This is, the activation of some brain areas that are not necessarily required in order to succed in the task. They have interpreted this suggestion specifically because the Amygdala and anterior STG do not distort the ability of participants to detect target stimuli (Kiehl et. al. 2005). Our results showed activation of the right STG in Z vs. G and N vs. G contrasts, but are in the same contrasts for the left STG. With regard to the difference in the left STG activations, when basic conditions are contrasted gave only NG vs G and N vs G. This supports the suggested hypothesis of the “adaptive reflexive processing” of Kiehl’s paper (2005) for Novel signals in the left STG.

Moreover, by using the contrasts, taking into account the immediately previous context, the Right STG is activated differently in all cases of contrast tested. But the Left STG is activated differently in most cases (Z.G vs. G.G, NG.G vs. G.G, and N.G vs. G.G) except N.G vs. Z.G and NG.G vs. Z.G contrasts. These different results show that there are no activation differences when compared to NG or N conditions with Z condition followed later by the G condition. This may show that the “adaptive reflexive processing” for this area showed that the NG or N is taking on a different role at recognizing Novel stimuli independently of the Goal or Non-Goal condition. This may be an extension of previous experiments because both local probabilities are different (G at local probability of 62.5 %) and the Goal is mixed with new stimuli (NG local probability of 12.5 %).

On the other hand, the number subtraction does not evoke the activation of the STG whereas number addition evokes its activation (Hamid, Yusoff, Mukari & Mohamad, 2011). According to the present results STG is activated in the contextual control of attention and that would mean that the subtraction on Hamid’s experiment may be explained partially as a consequence of the previous context.

Overall, the STG as a part of the TPJ would match the reflexive adaptive processing of Kiehl (2005) and number addition of Hamid (2011) into the framework of plasticity in the different routes of brain processing activity in the SDN and GDN for control of attention of Corbetta & Shulman (2002)

## Subcortical activations.

As a result of the auditory experiment, the TTG was considered in the fMRI analysis. This region possibly extends the visual reorienting model of Corbetta and colleagues to the auditory modality, but more analysis should be done to assure that this model may be extended to other modalities, for example an importante extension may be to model the subcortical areas inside the model. Therefore, having the advantage that the subcortical regions were compared, according to the literature, the Thalamus pulvinar is believed to channel the goal-driven network (Shipp, 2004). Wróbel and colleagues found a greater amplitude in the Thalamuspulvinar in the beta frequency for cats in visual discrimination than in auditory cues (Wróbel, Ghazaryan, Bekisz, Bogdan & Kamiński, 2007). The pulvinar – FEF connection with diffusion tensor imaging has also been proven (Leh, Chakravarty & Ptito, 2008), and a recent study suggested that the oscillatory activity of the Thalamus pulvinar may influence cortical processing in the visual cortex (Saalman & Kastner, 2011). Therefore an analysis can be carried out by the present experiment with regard to auditory modality.

In the present results, the thalamus pulvinar showed significant differences in the auditory number parity decision task. The Thalamuspulvinar at the Z vs G contrast showed activations that were relatively greater to G (no bias to FEF and Z.G vs G.G biased to G.G), N vs G contrast biased to G (no bias to FEF) and NG vs G contrast biased to NG with a bias to FEF in the fMRI analysis. Also, the Thalamus Pulvinar did not show significant differences in the N.G vs G.G contrast, *i.e.* it is not supporting a funnel activation in the Novel previous context. However, along FEF, the Thalamuspulvinar is biased to NG.G when compared with N.G condition and biased to NG.G as well when compared to Z.G condition.

Therefore, these results, although they do not provide evidence of different prior contextual effects of Goal and Novel trials, do support the idea that the right thalamus pulvinar has a role in the regulation of Novel signals in Goal and Non-Goal tasks in auditory tasks. This is the mixture of Novel and Goal, which may be influencing the Novel in the top-down mechanism for both the simultaneous NG event and the sequence of the stimulus given by the context. In other words, this is reorienting of attention.

## Use in multitasking modelling

Results in supplemental material confirms fMRI and EEG maybe used splited, although they did not abibe to be combined to strengh evidence of different prior contextual effects of Goal and Novel trials, do support the idea that attention was seen in EEG as well as fMRI main results. Therefore mulstitasking can be seen in EEG and fMRI form the same task. Moreover, a further work maybe to work with a visual experiment, where current noise would not affect as here was shown for EEG recordings.


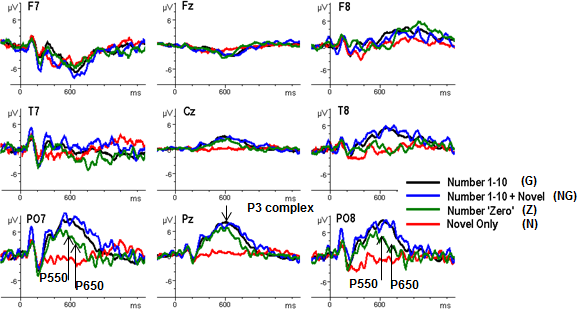


*Figure S1* Grand average ERP waveforms for parity decision Goal stimuli (G), simultaneous Novel and Goal stimuli (NG) as well trials on which no response was required, *i.e.* Zero (Z) and Novel (N) stimuli. The P3 complex appears to consist of distinct deflections occurring at 550 and 650 ms. The parietal P550 deflection dissociates on the basis of number versus novel stimuli whereas the P650 deflection dissociates on the basis of response or no response, most clearly at T8.


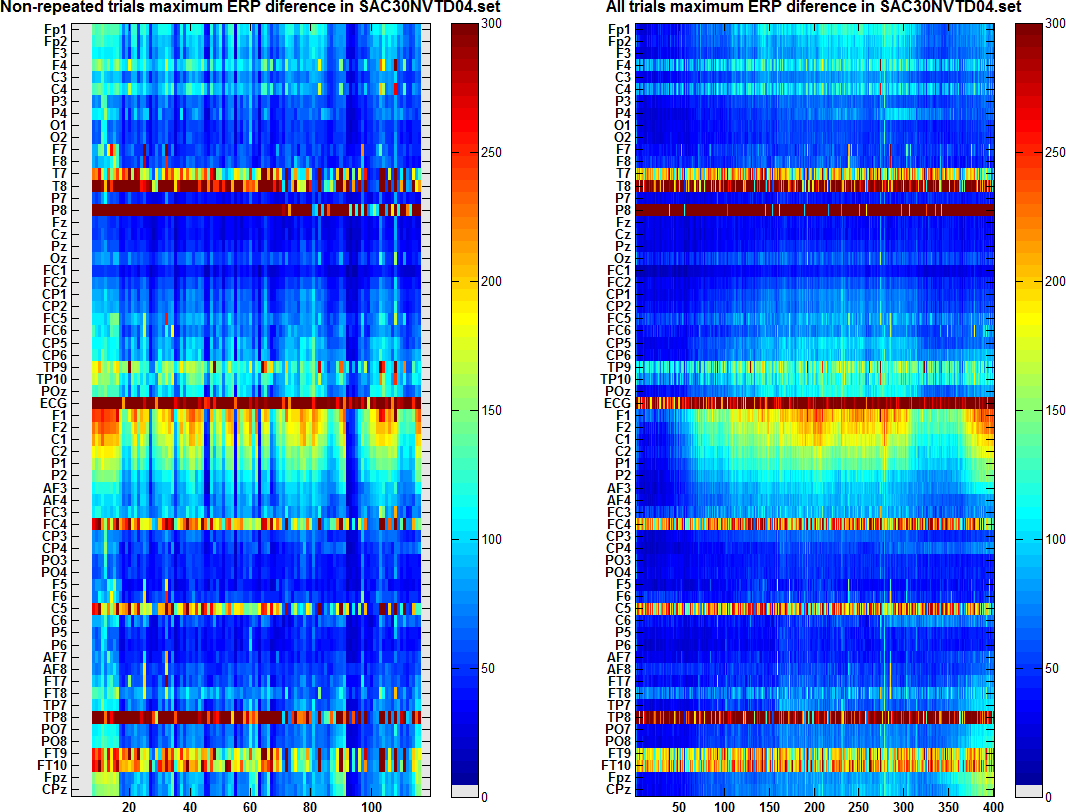


*Figure S2* Time difference of maximum EEG difference between the amplitudes of filtered and raw EEG data across channels from 0 to 400 ms after auditory onset stimulus in the participant labelled with fMRI04. On the left: trials grouped by codes. First numbers from 0 to 10 are shown, and later Novel events are shown. On the right: successive 400 trials.


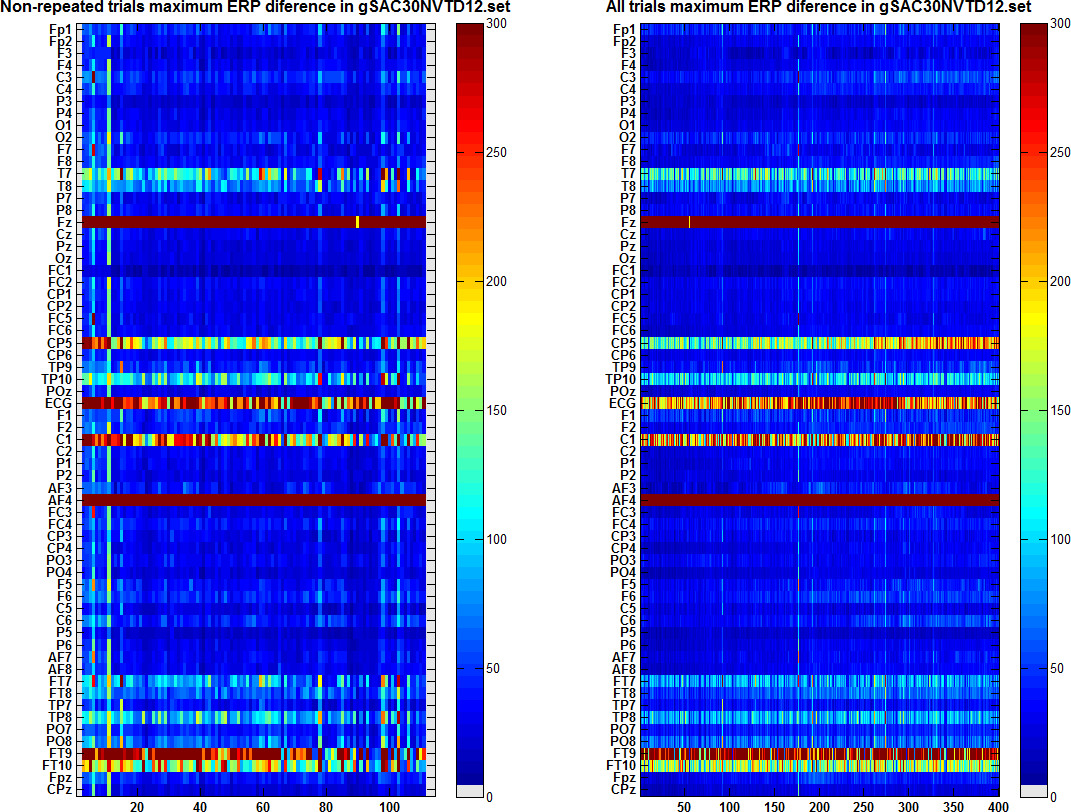


*Figure S3* Channel noise of maximum EEG difference between the amplitudes of filtered and raw EEG data across channels from 0 to 400 ms after auditory onset stimulus in the participant labelled with fMRI12. On the left: trials grouped by codes. First numbers from 0 to 10 are shown and later Novel events are shown. On the right: successive 400 trials.


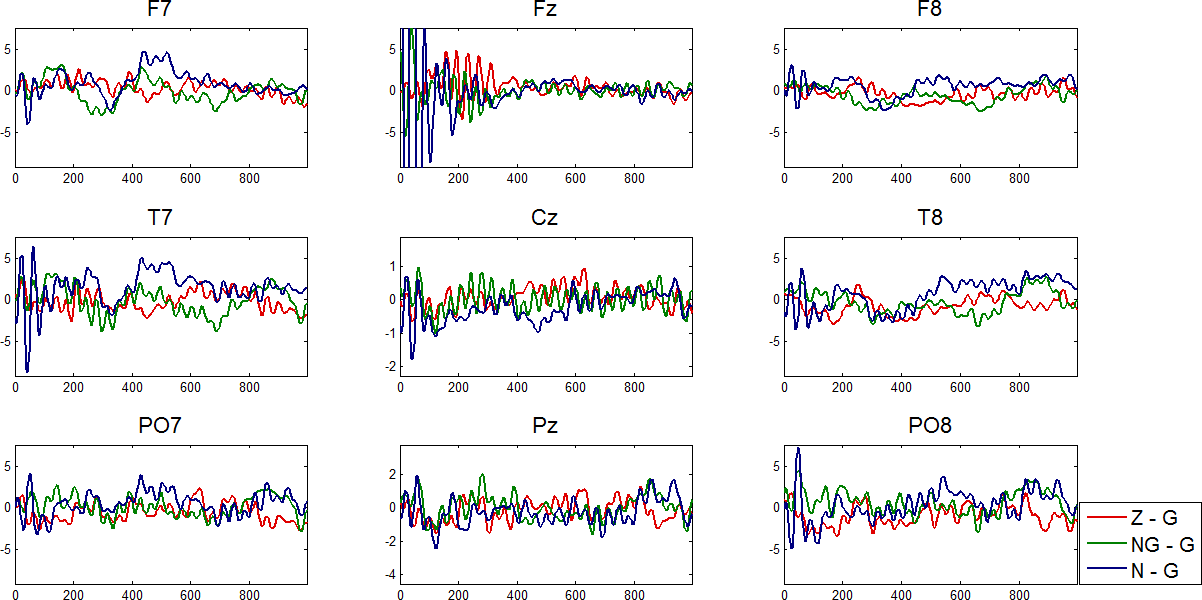


*Figure S4* Subtraction of the ERPs of the standard Goal stimuli from the Zero, simultaneous Novel and Goal, and Novel stimuli in few electrodes. Note that the amplitude scales in Cz and Pz are respectively four and two times that scales in the other electrodes.

## fMRI contrasts

The purpose of this experiment was to determine if the slowing of responses to simultaneous number and novel stimuli (NG) was associated with the activation of the stimulus driven attention network without activation of the goal driven system. The Novel only condition (N) was introduced to allow visualisation of an attention orienting response, assumed to involve activation of the stimulus and goal driven systems but also involving withholding a button press. The zero (Z) condition was introduced in an attempt to visualise the effects of withholding a response in the novel condition.

When the classical contrasts between N, Z, NG conditions and the G condition were carried out in all the participants, most of the N vs G contrast did not show relatively different activation and as well as the NG vs G did not show ventral areas with relatively different activation (detailed results of these analyses are not presented here). Therefore, these relatively different activations in all the participants were not supporting Corbetta and Shulman SDN and GDN in a cortical network for control of attention [5]. In this way, the detailed analysis is presented for the behavioural ‘distracted’ participants (n = 6). Tables S1 to S3 list the brain areas and their equivalent Brodmann Areas (BA) activated when the N, NG, and Z conditions were contrasted with the Goal (G) condition.

First, Figure S5 and Table S1 show and list the contrast between Zero (Z) and Goal (G) conditions, respectively. According to the results, there is no support for any brain areas activated with both positive and negative contrasts and also with different BAs. Both hemispheres in frontal, temporal and occipital areas showed differences, while in the parietal lobe only the left hemisphere showed significant differences. The Left and Right Superior Temporal Gyrus (R STG) showed differences and also the Right Superior Frontal Gyrus (R SFG), which is consistent with the stimulus-driven control proposed by (5); these differences are positively biased to the Z condition. On the other hand, although there were no differences in the Inferior Parietal Lobule (IPL), there are no differences in the left and right Brodmann Area 8 (Frontal Eye Field, FEF) and the expected different activation for IPs is not shown. Therefore, the brain areas do not show clearly the goal-driven control of attention proposed by [7], but these brain areas are consistent with the different frontal activity in BA 6 hypothesized for response inhibition in Go/No Go tasks [61].

Table S2 Brain areas and statistical results of Orienting Group (n = 6) with p < .03 (uncorrected) and 1055 voxels activated for Zeros vs Goal conditions: Z vs G


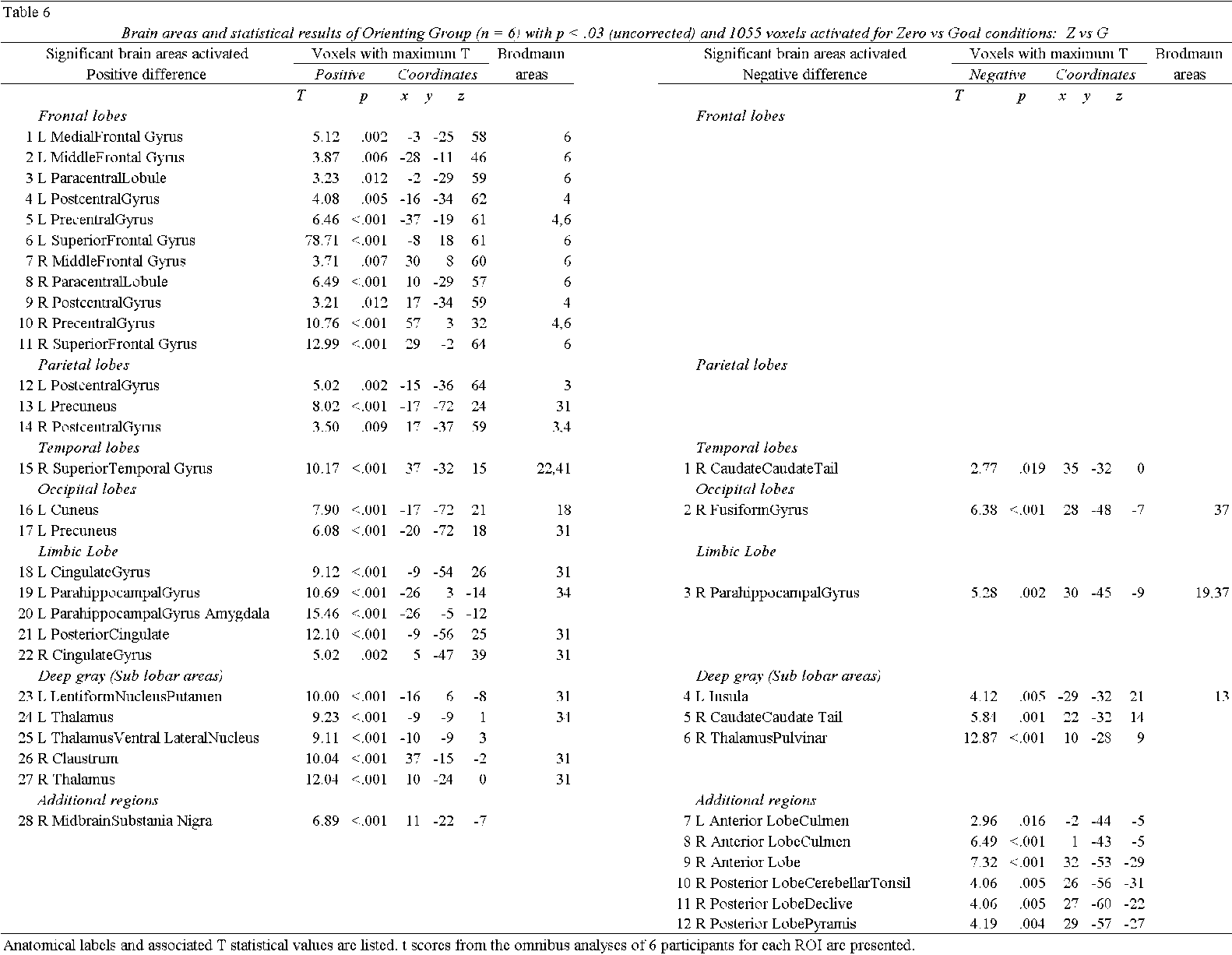


On the other hand, the primary motor area and the Precentral Gyrus in BAs 4 and 6 (in the left and right hemisphere) exhibited significant positive activation biased to the Z condition (see Table S1). This supports the hypothesis of different motor area activation due to the high Goal probability (62.5 %) over the Zero probability (12.5 %). Moreover, the Caudate Tail and Right Fusiform Gyrus (BA 37) are activated particularly in the G condition and this suggests a visual activation going from the lower part Temporal Lobe, which can be explained by a projection from the Caudate Tail to the Superior Colliculus, and allows visual information to evoke saccadic eye movements [62] and it is found stronger for high-value objects [16] and that possibly activates the Right Fusiform Gyrus.

The results of the contrasts between Novel (N) and Goal (G) conditions are shown on middle part of Figure 3 and tabulated in Table S2. Both hemispheres in frontal, temporal and limbic brain areas showed differences, while in the temporal lobe only the left hemisphere showed a difference, whereas in the parietal lobe only the right hemisphere showed significant differences. According to the results, only the Right STG in the BA 22 is activated with both positive and negative contrasts. Also, the Right Precentral Gyrus, Right SFG and the Right STG are activated with positive and negative contrasts but in different Brodmann Areas (see highlighted results in Table S2). The STG showed positive differences in the Left (BA 6) and Right (BA 6). The Left Inferior Frontal Gyrus (IFG) in BA 47 and Right IFG (BA 47) showed positive differences and other parts of the Right IFG showed negative differences. The Right IPL showed negative differences (BA 40). The Right Middle Frontal Gyrus (MFG) showed negative differences (BA 4). The Left and Right STG showed positive differences while the Right STG showed negative differences, which is consistent with regions involved in the stimulus-driven control network proposed by Corbetta and Shulman [7] and part of the reorienting network proposed by Corbetta and colleagues [19]. There are also negative differences observed in the IPL (BA 40), which is consistent with Krall and colleagues’ interpretation of the function of the anterior R TPJ function in attention to Corbetta’s model [62]. On the other hand, no differences were observed in the Brodmann Area 8 (FEF). Therefore having no clear activations in the IPs (sought in BA 7, 19, 39 and 40), there is no clear evidence of differences in the goal-driven control of attention proposed by Corbetta & Shulman [7]. The involvement of the GDN and SDN for the orienting response in the hypothesis H1 has been supported for the stimulus-driven network.


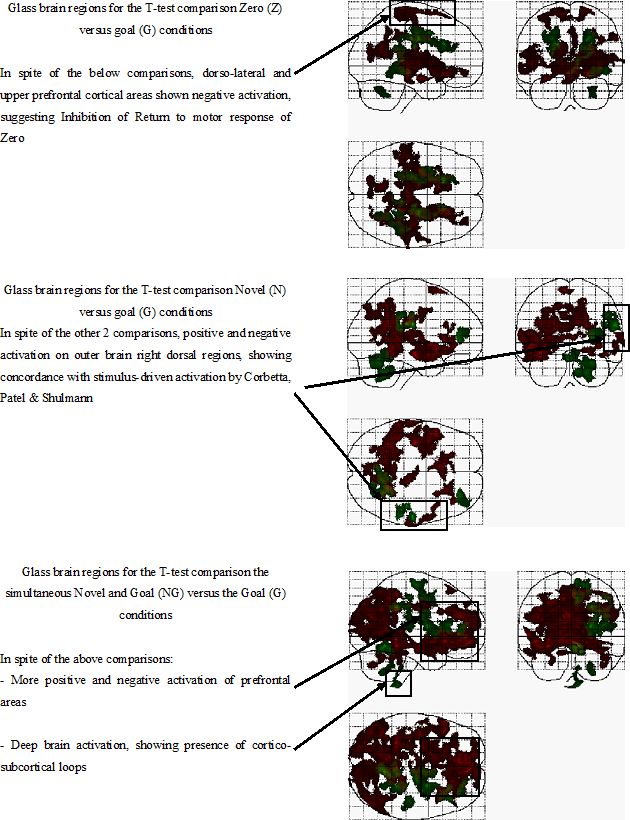


*Figure S5* Glass brain regions for the T-test comparisons: Zero (Z) versus goal (G) conditions. Novel (N) versus goal (G) conditions and Novel and Goal (NG) versus the Goal (G) conditions. Red are positive T-values, Green are negative T-values and Yellow are both negative and positive overlapping}

Bearing in mind the result of the other contrasts, these positive and negative differences in the frontal, parietal and temporal lobes will be considered in the discussion. The high Goal probability (62.5 %) over the Novel probability (12.5 %), produced activations in the primary motor area and the Precentral Gyrus (in the left and right hemisphere) exhibited significant positive activation dorsally biased (BA 4) to the N condition and ventrally biased (BA 43) to the G condition (see Table S2). Therefore, the different motor activation of hypothesis H5 is visualized when Goal and Novel only conditions are contrasted.

The contrasts between simultaneous Novel and Goal (NG) and Goal (G) conditions are shown Figure 3 at the bottom and tabulated on Table S3. Both hemispheres in frontal, temporal, parietal, occipital and limbic brain areas showed differences. According to the analysis, only the Right Medial Temporal Gyrus in the BA 10 and the Right Cingulate Gyrus in the BA 24 are activated in both positive and negative contrasts (see highlighted results in Table S3). Also, the Right Precentral Gyrus, the Right SFG, the Right STG, and the Right Anterior Cingulate Cortex have activations in positive and negative contrasts but in different Brodmann Areas. Brain differences are biased to the NG condition in the Left STG, in the Left and Right IFG, and the Left and Right Medial Frontal Gyrus, which is consistent with stimulusdriven control proposed by Corbetta& Shulman [7]. Also, important for hypothesis H1 are the differences in activation in the Right IPL, which is consistent with goal-driven control proposed by Corbetta & Shulman [7], although this is biased for the Goal condition. Therefore, the additional activation of the SDN has not been found in G vs NG conditions. However, it is important to remember that these participants showed slower RTs in the NG condition, in part due to the effect of orienting response matched with conflict monitoring. In this way, considering the activation of both sides of the Anterior Cingulate Cortex in positive and negative contrasts (Table S3), this matches with areas of activation observed in conflict monitoring [65]. This is addressed in the discussion along with the other contrasts.

Also, significant differences were found in the primary motor area, the Precentral Gyrus (in both hemispheres) between G and NG conditions (see Table S3). Differences in the left hemisphere were biased frontally to the NG condition and on the right hemisphere to the G condition. This is supporting the idea of the different motor area activation due to that the high Goal probability (62.5 %) over the simultaneous Novel and Goal probability (12.5%).

Table S3 Brain areas and statistical results of Orienting Group (n = 6) with p < .03 (uncorrected) and 1055 voxels activated for simultaneous Novel and Goal vs Goal conditions : NG vs G


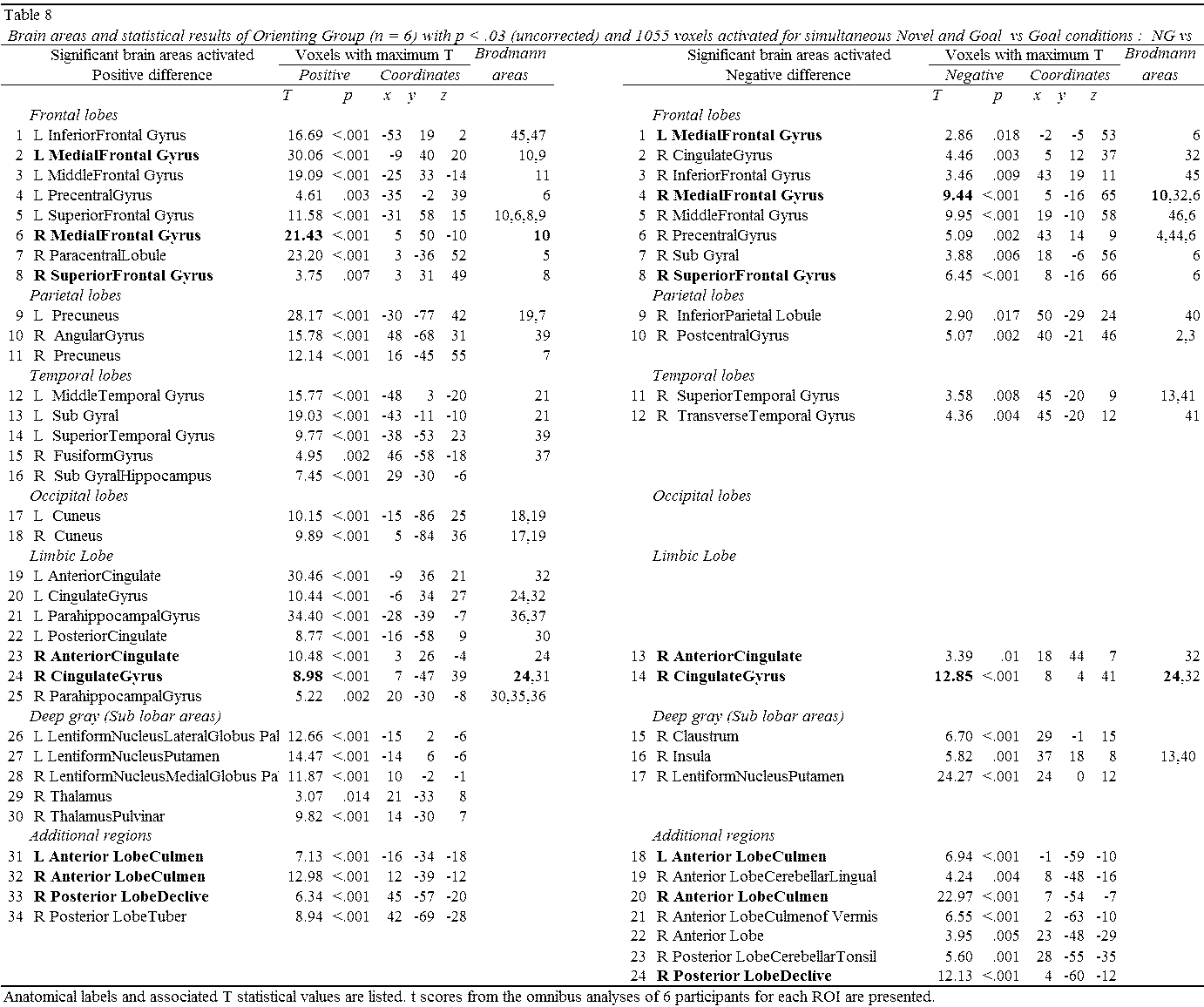


## fMRI for ‘distracted’ participants showed differences in activation in left and right brain areas in the attention model (H1).

The graphic in Figure S6 shows that N vs. G show more right parietal lateralized differences while Z vs. G showed left parietal activations. These results are consistent with Corbetta and Shulman’s model of attention [7] and with the parietal sources of attention for P3a suggested by Polich [66]. Also, NG vs. G has differences spread in several brain areas.


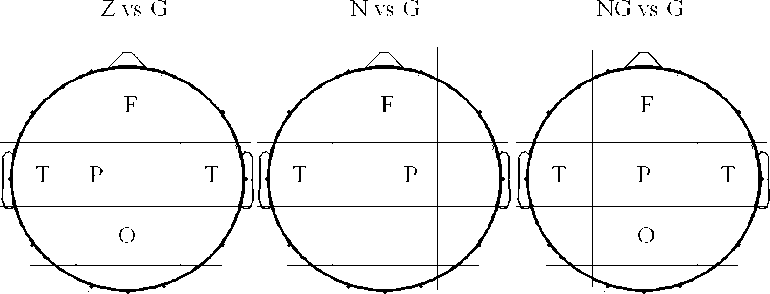


*Figure S6* Comparison of the different brain regions for the contrasts Z vs. G, N vs. G and NG vs. G conditions.

The results of this work are discussed in the context of the model of attention, proposed by Corbetta: the areas identified in this model are the Left and Right FEF in Brodmann Area 8, the Left and Right MFG, the Left and Right IFG, the Left and Right IPL, the Left and Right STG, and the Left and IntraParietal Sulcus (L IPs and R IPs) [7], [19]

### N vs. G contrast, considered in the context of Corbetta’s model of reorienting of attention.

The results of the did show different activation of the MFG for contrast N vs G and G vs N but they did not show clear differences for the R IPs or close brain areas. Although these results are not clearly consistent with the reorienting of attention per Corbetta and colleagues [19], they support the stimulus driven attention network of Corbetta and Shulman [7]. Brain Areas involved in the orienting response (i.e. GD+SD) were visualized when Goal and Novel only conditions were contrasted and there were found reduced motor activation and enhanced frontal inhibition systems activation.

Moreover, another area related to auditory stimulus processing in Kiehl’s results were the left Transverse Temporal Gyri (TTG) which was activated for Goal over Novel stimuli (p <.001 FWE) with local probabilities for Goal and Novel of 10 % and 10 %, respectively [16]. To this author’s knowledge, there are no articles focusing on Novel stimulus to discuss this TTG area at different local probabilities such as in the present experiment. However, in the two-oddball tasks the TTG appears biased to the infrequent target. For example, Stevens and colleagues found this bias, having 12 standard non-goal stimuli between the infrequent target [67]. In our results, the TTG appears more activated in both hemispheres biased to the N condition over the G condition. A possible reason may be the local probability of the N condition 12.5% vs. the local probability of the G condition of 62.5%. Therefore, the present results added to the literature, suggesting that the auditory cortex is modulated by the local probability.

In the present results, the more dorsal R SFG appeared activated on both contrasts instead of the R FEF and this would change not only the top-down control but also the stimulus-driven network changing the R FEF to the Right SFG. However, this may simply be a consequence of normalisation not working as well due here to the small population of participants.

### NG vs. G contrast and the Corbetta model of reorienting of attention.

Extending this discussion of the attention model and the auditory task, taking into account that the results are consistent with the activation of the stimulus driven attention network of Corbetta and Shulman [7]. Although, the positive contrast results are not exactly consistent with the reorienting of attention of Corbetta and colleagues [19], activation in Brodmann Areas 7, 19 and 39 are possibly consistent with activation of the R IPs. On the other hand, the negative contrast (biased to G condition) showed a different activation in the Brodmann Area 40 and the SPL indicating a weak relationship with the R IPs and the TTG indicating a greater activation of the auditory cortex for the G condition, corroborating Kiehl’s results for a Goal condition [16]. Moreover, this negative contrast is consistent with the reorienting of attention of Corbetta and colleagues [19]. These interpretations suggest that the NG condition is possibly evoking a similar pattern to orienting of attention, while the IPs is suppressed, possibly due to an auditory cortical bias for the G condition. This possibly affects R MFG having R Medial Frontal Gyrus instead.

### Z vs. G contrast and the Corbetta model of reorienting of attention.

Finally, concluding this relation between the attention model and the auditory task, taking into account the results of the positive contrast results, however, are not consistent with the reorienting of attention of Corbetta and colleagues [19]. Although several areas of the right hemisphere were with relatively greater to the Z condition, the FEF and IPL are not clearly activated or deactivated. These interpretations suggest that the Z condition is evoking a similar pattern to control of attention without difference of the Transverse Temporal Gyrus for the Numbers in Z and G condition, i.e. the stimulus driven control of attention in the model of Corbetta and Shulman [7]. Response inhibition were supported by frontal areas in the, Goal vs. Zero conditions, therefore this supports the idea of the capacity to inhibite unsuitable actions in this task for this group of participants.

The results of the Table S1 did show different activation of the MFG for contrast N vs G and G vs N but they did not show clear differences for the R IPs or close brain areas. Although these results are not clearly consistent with the reorienting of attention per Corbetta and colleagues [19], they support the stimulus driven attention network of Corbetta and Shulman [7]. Brain Areas involved in the orienting response (i.e. GD+SD) were visualized when Goal and Novel only conditions were contrasted and there were found reduced motor activation and enhanced frontal inhibition systems activation.
